# Supplementary material for: Membrane-mediated interaction of non-conventional snake three-finger toxins with nicotinic acetylcholine receptors
Source: Commun Biol. 2022 Dec 7;5:1344. doi: 10.1038/s42003-022-04308-6 (PMC9729238; doi:10.1038/s42003-022-04308-6)
Supplement: Supplementary file 2 — Supplementary Information [file 42003_2022_4308_MOESM2_ESM.pdf]

Supporting information

Membrane-mediated interaction of non-conventional snake three-finger toxins with nicotinic acetylcholine receptors

Zakhar O. Shenkarev[1,2,*], Yuri M. Chesnokov[3,4,*], Maxim M. Zaigraev[1,2,*], Anton O. Chugunov[1,2,5,*], Dmitrii S. Kulbatskii[1,*], Milita V. Kocharovskaya[1,2], Alexander S. Paramonov[1], Maxim L. Bychkov[1], Mikhail A. Shulepko[1], Dmitry E. Nolde[1,5], Roman A. Kamyshinsky[3,4], Evgeniy O. Yablokov[6], Alexey S. Ivanov[6], Mikhail P. Kirpichnikov[1,7], Ekaterina N. Lyukmanova[1,2,7,#]

[1]Shemyakin-Ovchinnikov Institute of Bioorganic Chemistry, Russian Academy of Sciences, Miklukho-Maklaya 16/10, Moscow, 117997, Russia

[2]Phystech School of Biological and Medical Physics, Moscow Institute of Physics and Technology (National Research University), Institutsky Lane 9, Dolgoprudny, Moscow Region, 141701, Russia

[3]National Research Center "Kurchatov Institute", Academic Kurchatov sq. 1, Moscow, 123182, Russia

[4]Shubnikov Institute of Crystallography of Federal Scientific Research Centre "Crystallography and Photonics" of Russian Academy of Sciences, Leninsky prospect 59, Moscow, 119333, Russia

[5]National Research University Higher School of Economics, Myasnitskaya str. 20, Moscow, 101000, Russia

[6]Institute of Biomedical Chemistry, Pogodinskaya 10k8, Moscow, 119121, Russia

[7]Interdisciplinary Scientific and Educational School of Moscow University «Molecular Technologies of the Living Systems and Synthetic Biology», Faculty of Biology, Lomonosov Moscow State University, Leninskie Gory, Moscow, 119234, Russia

* These authors contributed equally

#Correspondence to Ekaterina N. Lyukmanova, +7(903)2747912, ekaterina-lyukmanova@yandex.ru, Shemyakin-Ovchinnikov Institute of Bioorganic Chemistry, Russian Academy of Sciences, Miklukho-Maklaya str. 16/10, Moscow, 117997, Russia

Supplementary Figures

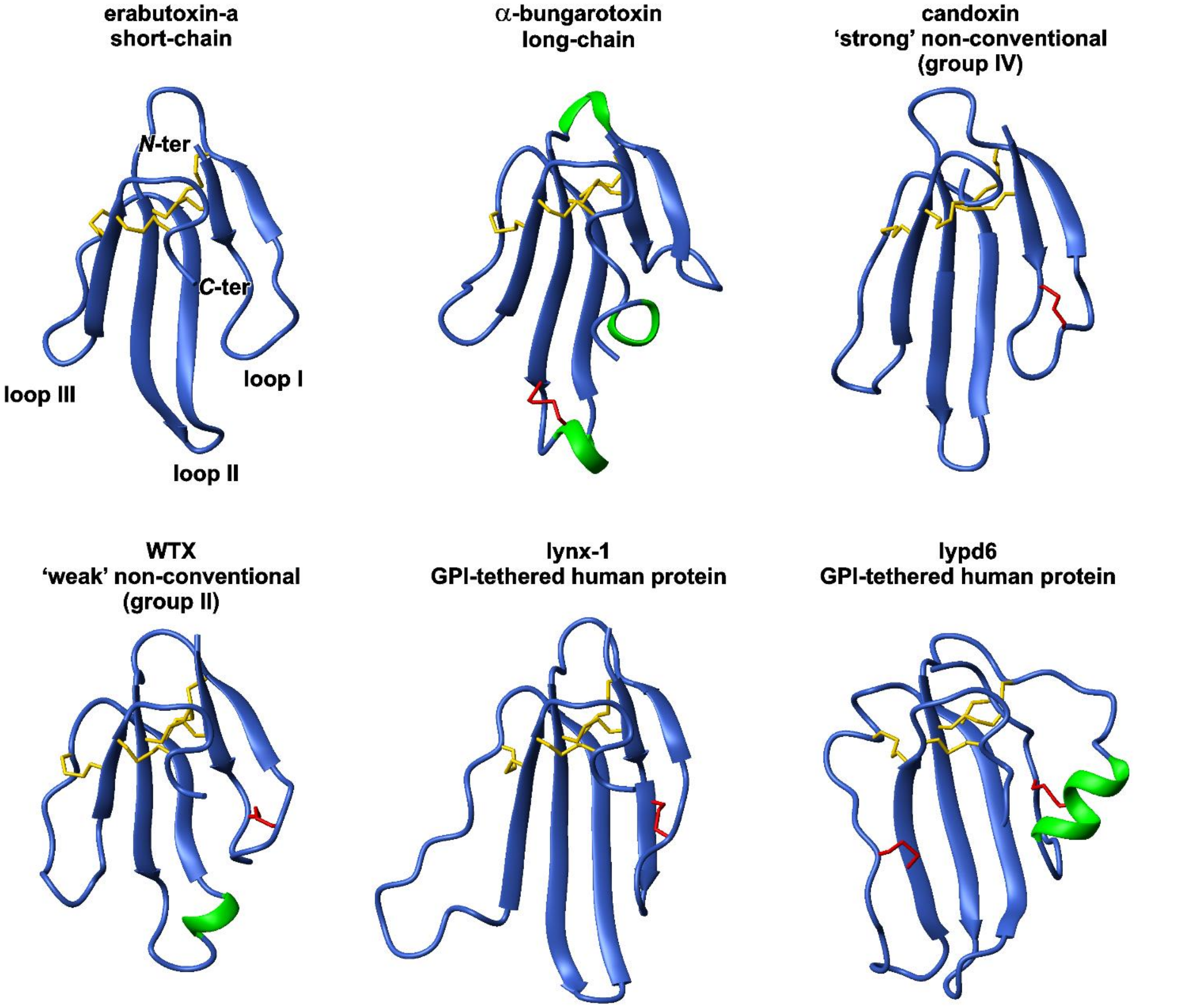

Supplementary Fig. 1. Comparison of spatial structures of three-finger α-neurotoxins from snake venom (short-chain erabutoxin-a, long-chain α-bungarotoxin, non-conventional candoxin and WTX) and water-soluble domains of human three-finger proteins Lynx1 and Lypd6. Four invariant disulfide bonds are colored in yellow. Additional disulfides in the loop regions are in red. The atomic coordinates were taken from the Protein Data Bank (PDB IDs 1QKD, 1KFH, 1JGK, 2MJ0, 2L03, 6IB6, respectively).

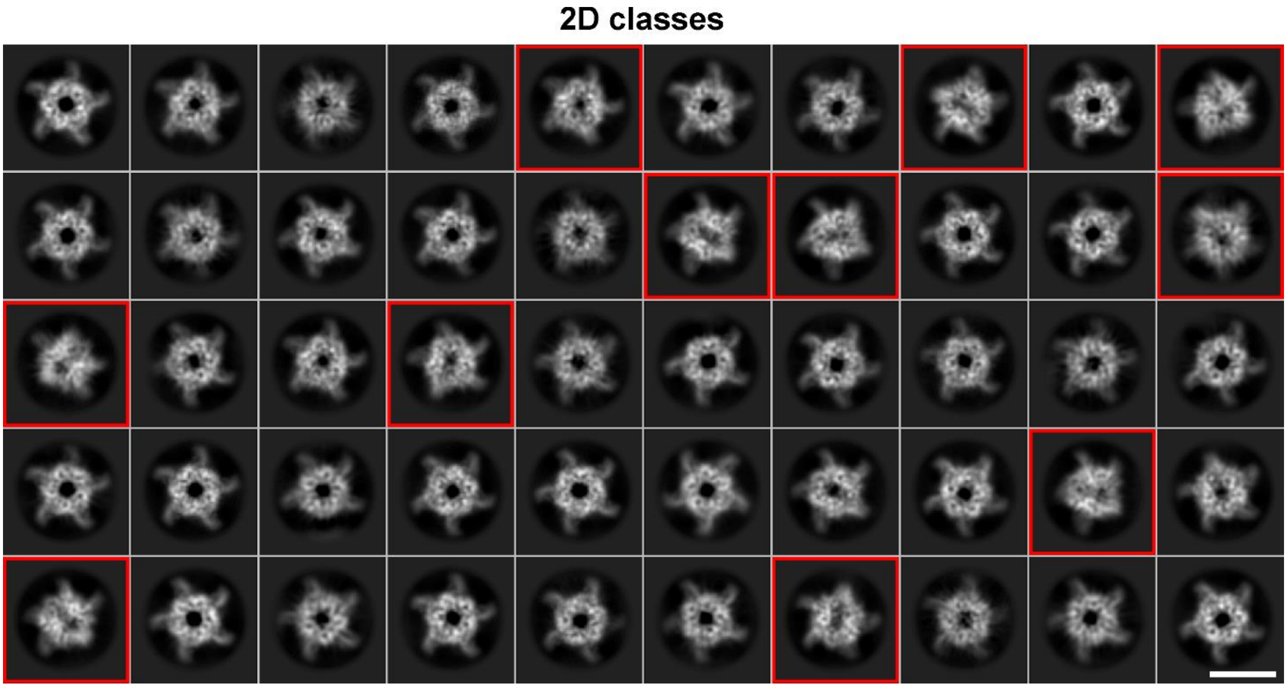

Supplementary Fig. 2. **2D classes of the α-Bgtx/α7-ECD complex**. The classes corresponding to tilted particles are highlighted. Scalebar is 10 nm.

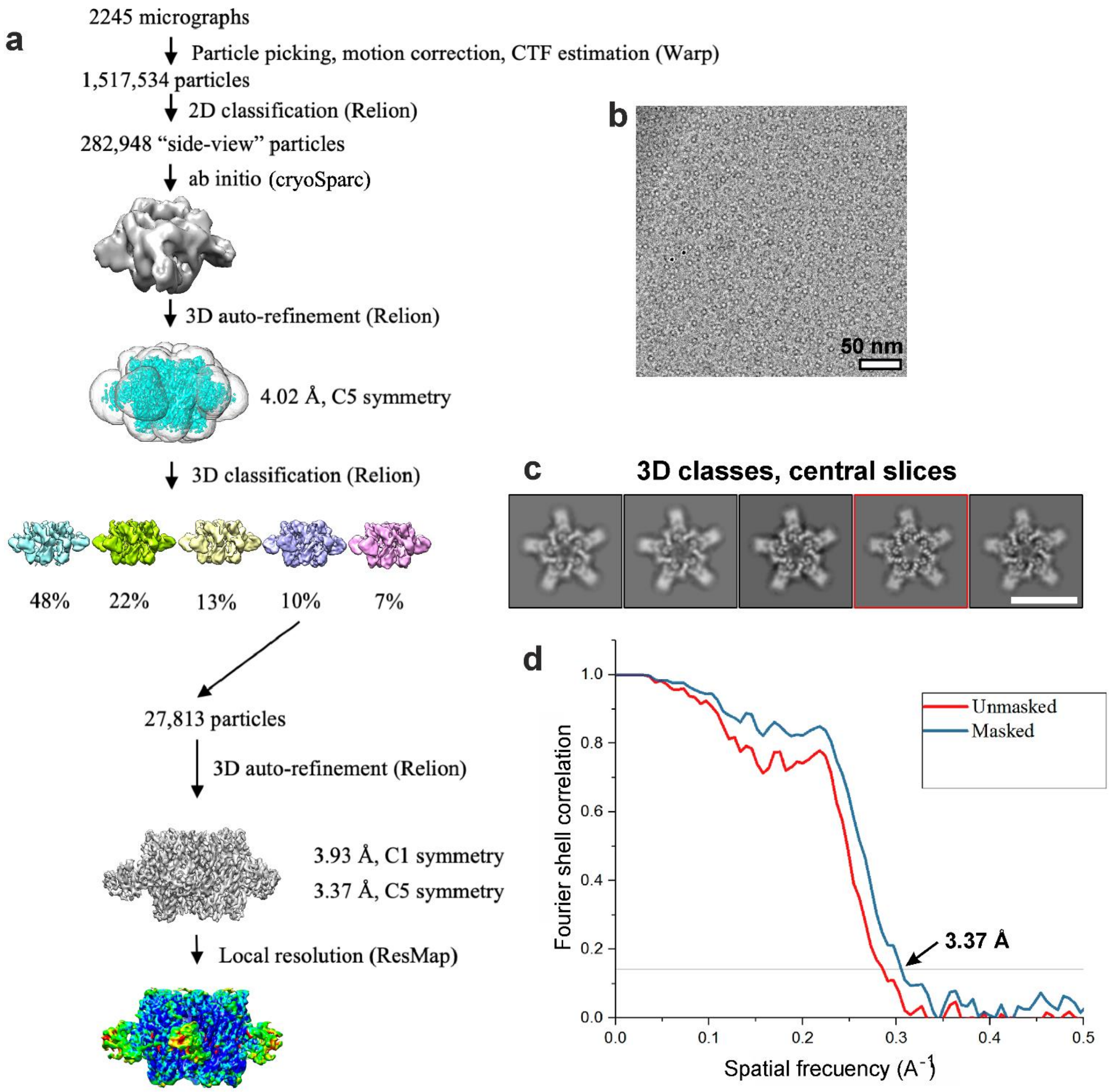

Supplementary Fig. 3. **Reconstruction of the 3D cryo-EM map for the α7-ECD/α-Bgtx complex**. **a** Overview of the image processing procedure. **b** Representative electron micrograph of the α7-ECD/α-Bgtx complex on the graphene oxide support film reveals mainly top-view orientation of the particles. **c** Central slices through 5 cryo-EM maps obtained from 3D classification, the best class is highlighted. Scalebar is 10 nm. **d** FSC (Fourier Shell Correlation) curve for masked (blue) and unmasked (red) map.

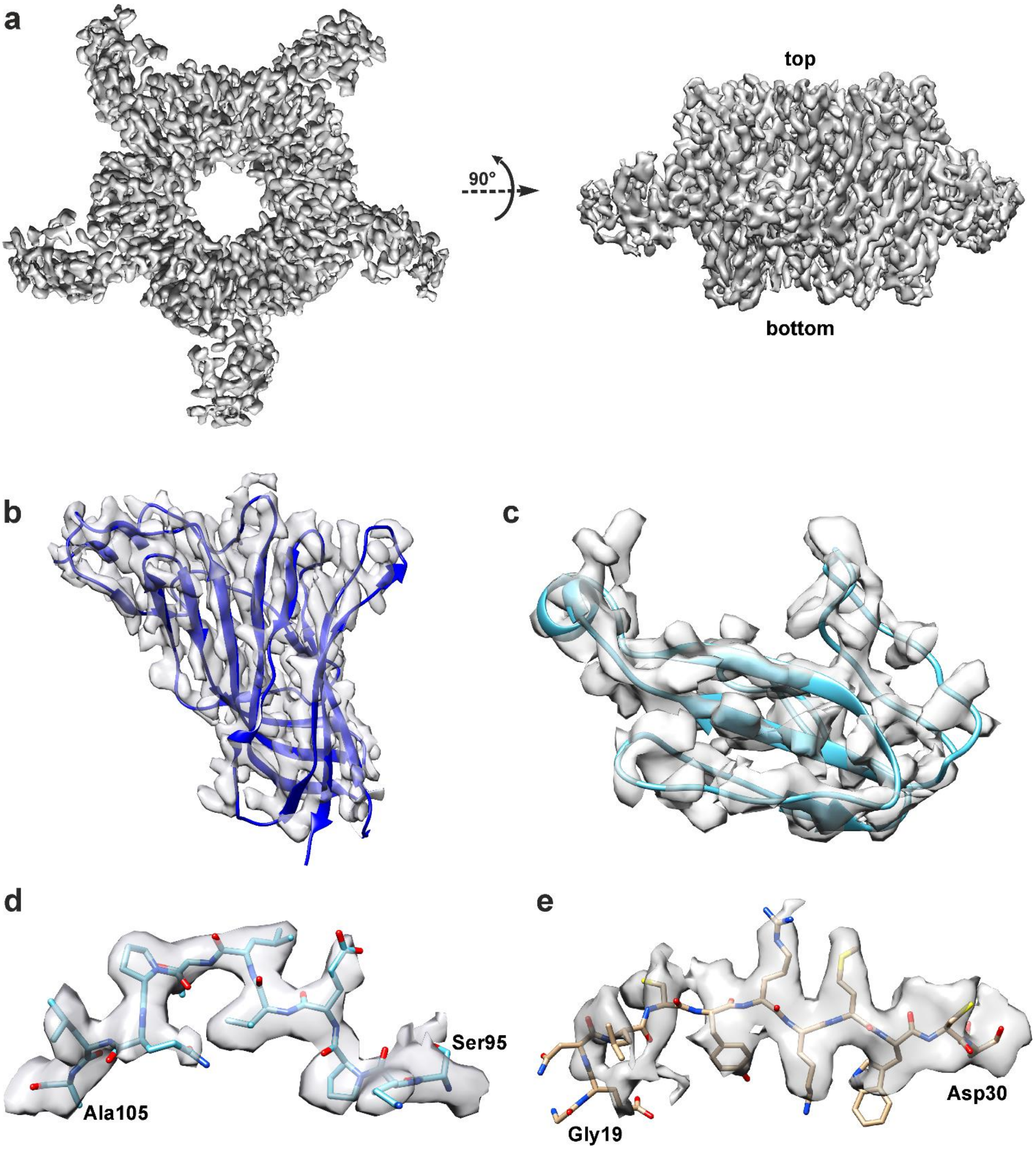

Supplementary Fig. 4. **Comparison of the obtained 3D cryo-EM map with crystal structure of the α7-ECD/α-Bgtx complex** [1]. **a** Top-view and side-view of the cryo-EM map. **b, c** Cryo-EM density map of the α7-ECD subunit (**b**) and α-Bgtx (**c**). Representative maps for regions are shown with the corresponding atomic model: α7-ECD (**d**) and α-Bgtx (**e**).

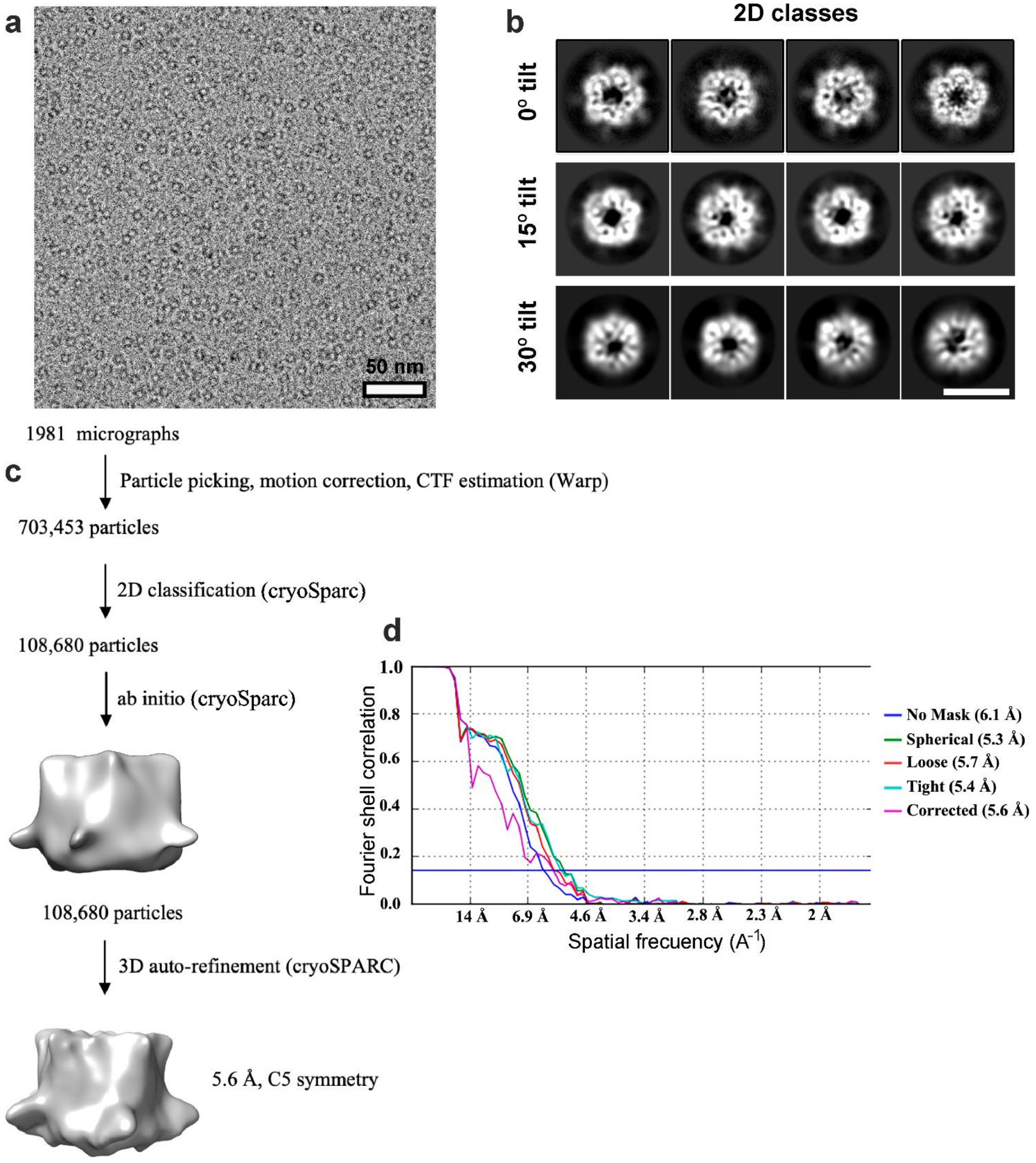

Supplementary Fig. 5. **Reconstruction of the 3D cryo-EM map for the α7-ECD/WTX complex. a** Typical cryo-EM image (bar is 50 nm). **b** Selected 2D classes obtained at 0°, 15°, and 30° tilt angles. Scalebar is 10 nm. **c** Overview of the image processing procedure. **d** FSC (Fourier Shell Correlation) curve generated in cryoSPARC.

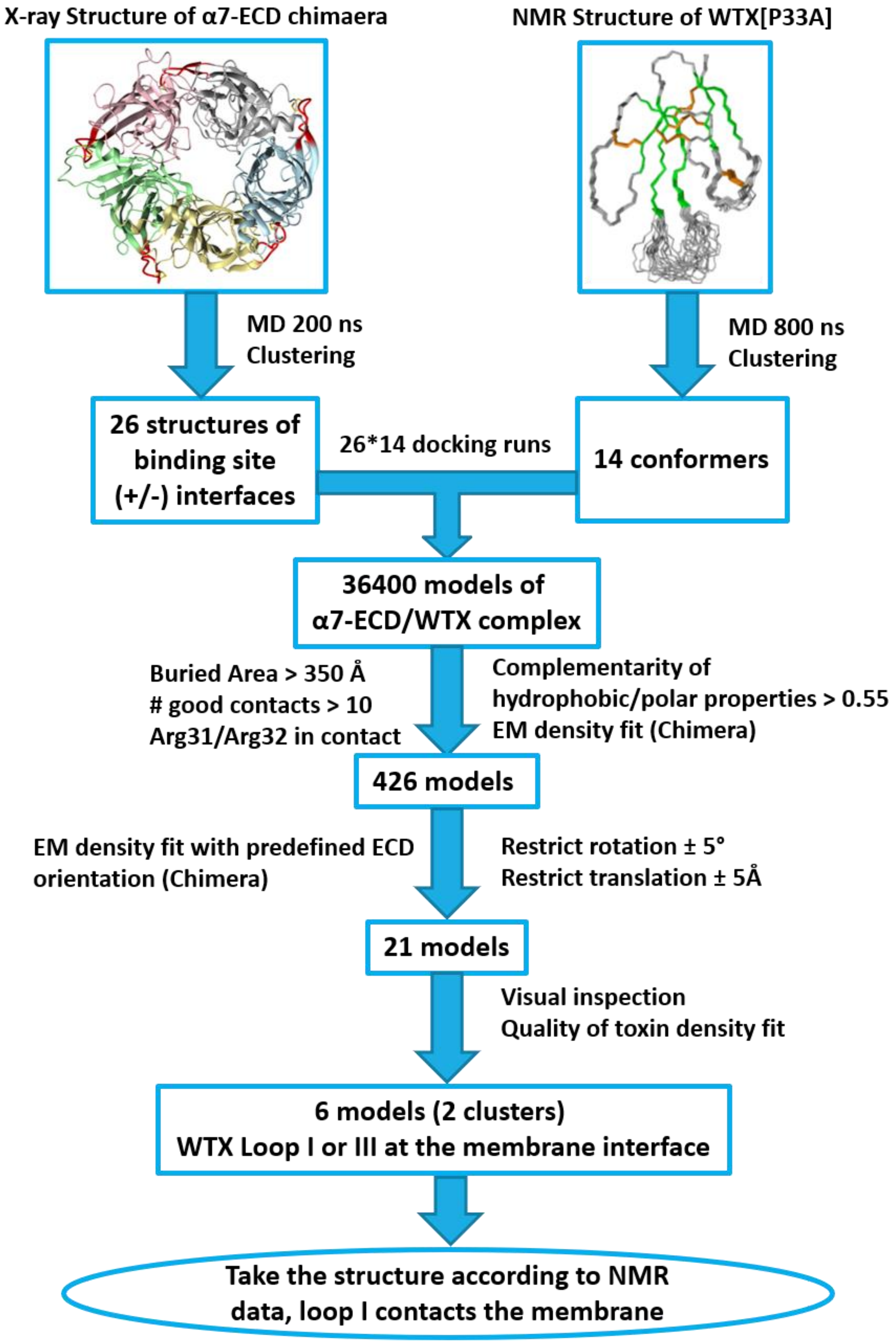

Supplementary Fig. 6. **Integration of cryo-EM, mutagenesis, NMR, and "ensemble" protein–protein docking for determination of the WTX/α7-ECD complex structure.**

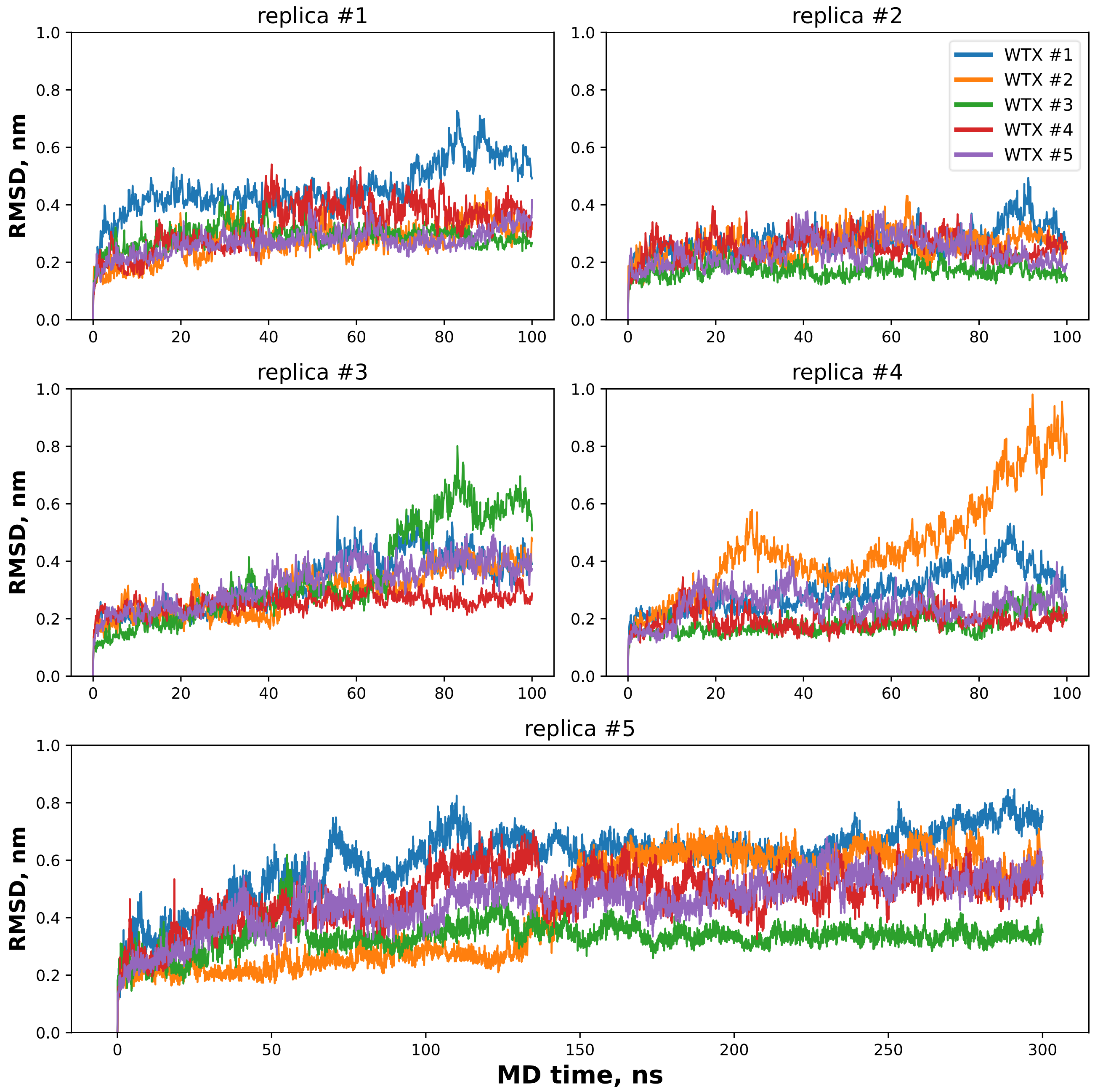

Supplementary Fig. 7. Plot for WTX RMSD from the initial position. RMSD values in nm for each WTX molecule in five calculated replicas during the whole time (100 or 300 ns) of molecular dynamics are shown. Replica #5 is the long (300 ns) production MD trajectory; replicas #1–4 are the short (100 ns) "checking the equilibrium" trajectories initiated from 0, 100, 200 and 300 ns of initial replica #5. Figures 4 and 5 in the main manuscript show the second WTX molecule (WTX #2 on the chart) from replica #5.

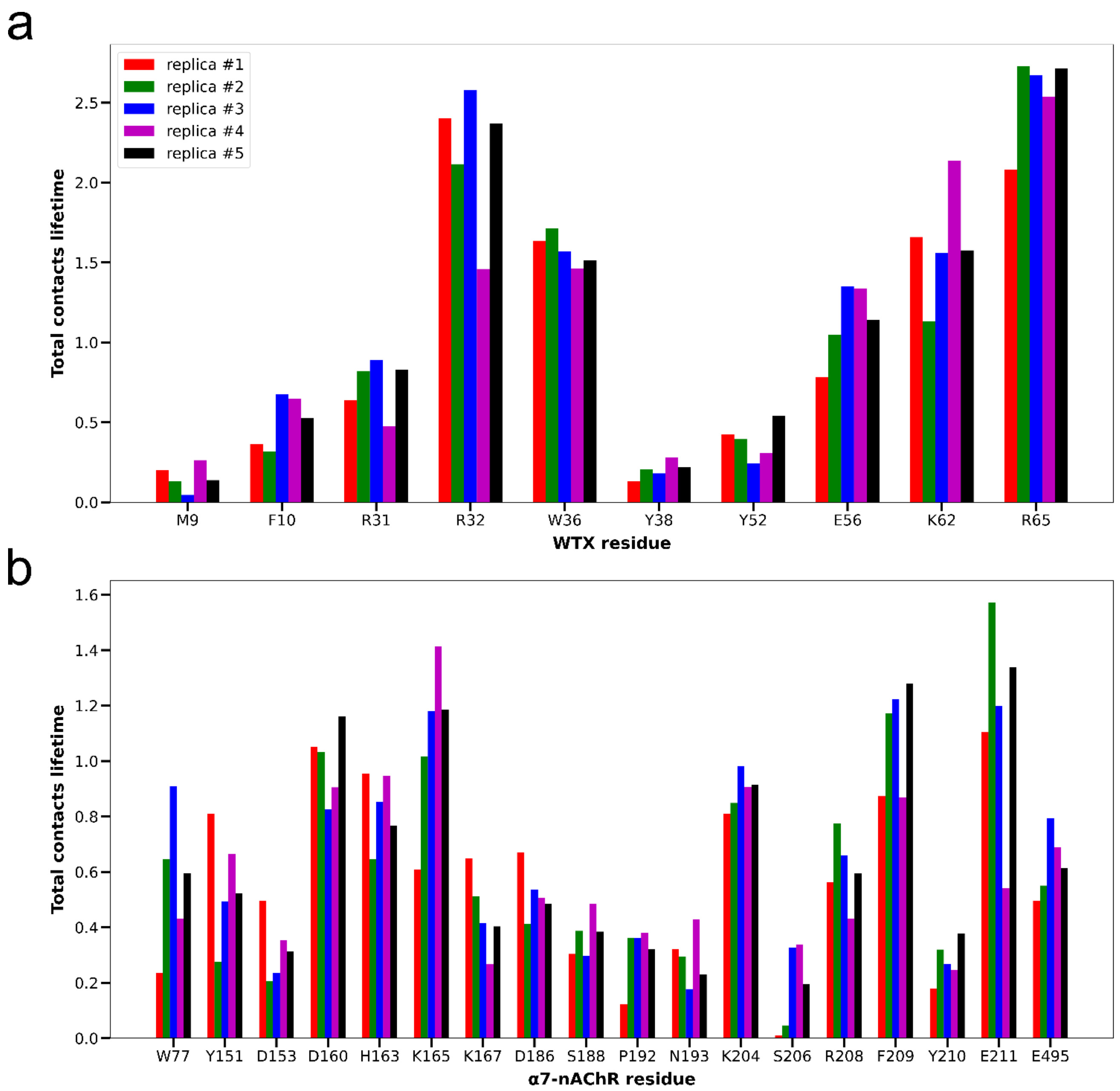

Supplementary Fig. 8. Total lifetime of intermolecular contacts for WTX (a) and α7-nAChR (b) residues in five MD replicas. Ionic, ion-dipole, π-cation, and stacking interactions and hydrogen bonds are included in total contacts lifetime. Replica #5 is the long (300 ns) production MD trajectory; replicas #1–4 is the short (100 ns) “checking the equilibrium” trajectories initiated from 0, 100, 200 and 300 ns of initial replica #5. For α7-nAChR residues, the lifetimes of contacts in any two replicas correlate with Pearson’s coefficient in the range of 0.55 - 0.95; for WTX residues, the correlation coefficients are in the range 0.85 - 0.98. This indicates the equilibrium state of the modeled WTX/α7-nAChR complex.

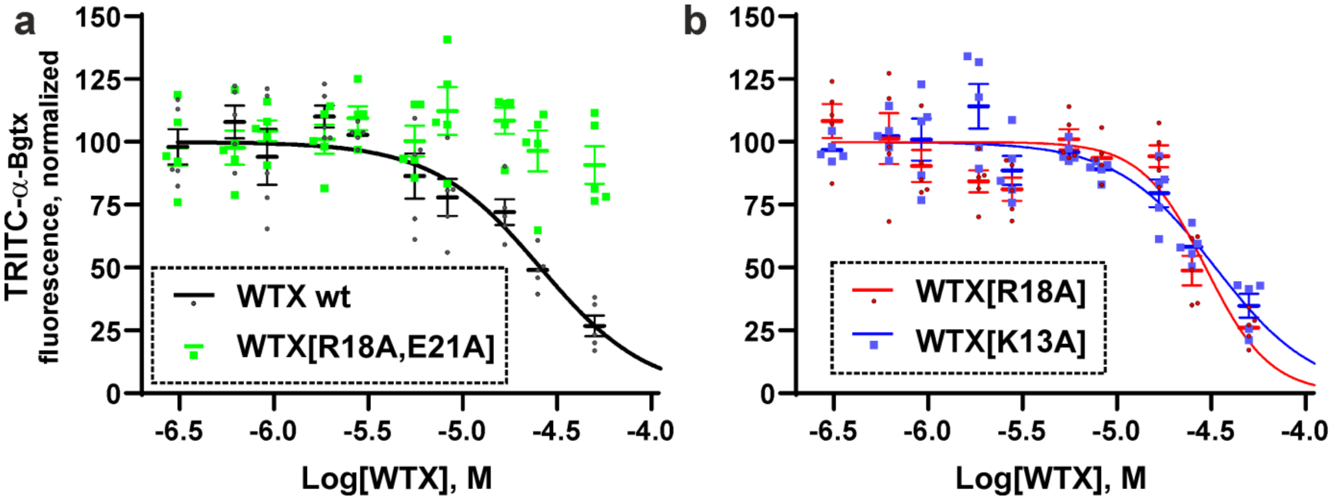

Supplementary Fig. 9. Competition of WTX and its mutants with TRITC-labelled α-Bgtx for binding to α7-nAChR expressed in HEK293 cells. Data were normalized to the TRITC-α-Bgtx-binding in absence of WTX and shown as mean ± SEM (n = 5 independent experiments). The Hill equation ($y = 100\%/(1 + ([Toxin]/IC_{50})^{nH})$) was fitted to normalized data.

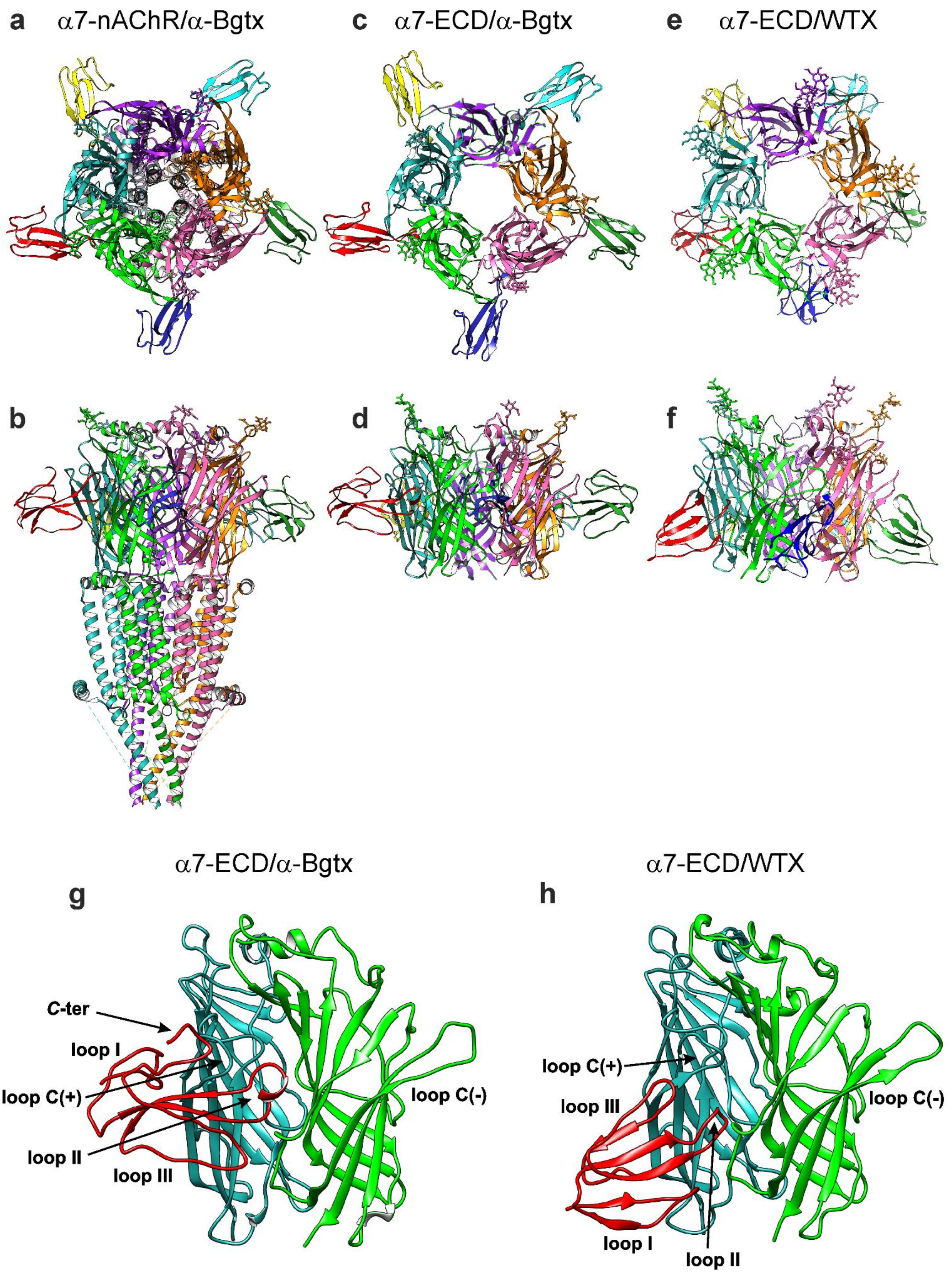

Supplementary Fig. 10. Comparison of the α7-nAChR/α-Bgtx (a,b, PDB ID: 7KOO [2]**), α - ECD/α-Bgtx (c,d,g, PDB ID: 4HQP** [1]**), and α7-ECD/WTX (e,f,h) structures**.

Non-conventional toxins. Orphan group II (WTX group, weak toxins)

```
P82935 NAJKA LTCLNCPEMFCGKFQICRNGEKICFKKLHQRRPLSWRYIRGCADTCPVGKPYEMIECCSTDKCNR 5
C1IC49 WALAE LTCLICPKKYCNQVHTCRNGENLCIKTFYEGNLLGKQFKRGCAATCPEARPREIVECCSRDKCNH 3
A2CKF6 BUNFA LTCLICPERYCQKVHTCRGEEKLCVKRFYDEKALGWRAKRGCAATCPEAKPKETVECCSTDKCNK 5
P85520 NAJOX LTCLICPEKYCNKVHTCRNGEKICFKKFDQRKLLGKRYIRGCAATCPEAKPREIVECCSTDKCNH 4
A2CKF7 BUNFA LTCLICPEKYCQKVHTCRDGENLCVKRFYEGKRFGKKYPRGCAATCPEAKPHEIVECCSTDKCNK 4
Q9YGI4 NAJAT LTCLNCPEMFCGKFQICRNGEKICFKKLHQRRPFSLRYIRGCAATCPETKPRDMVECCSTDRCNR 5
P01400 NAJME LTCLICPEKYCNKVHTCRNGENICFKRFYEGNLLGKRYPRGCAATCPEAKPREIVECCSTDKCNH 3
P01399 NAJHA LTCFNCPEVYCNRFHTCRNGEKICFKRFNERKLLGKRYTRGCAATCPVAKPREIVECCSTDRCNH 4
P01401 NAJHH LTCLICPEKYCNKVHTCRNGENQCFKRFNERKLLGKRYTRGCAATCPEAKPREIVECCTTDRCNK 4
P25680 NAJNI LRCLNCPEVFCRNFHTCRNGEKICFKRFDQRKLLGKRYTRGCAVTCPVAKPREIVECCSTDGCNR 3
O93422 NAJAT LTCLICPEKYCNKVHTCLNGEKICFKKYDQRKLLGKRYIRGCADTCPVRKPREIVECCSTDKCNH 3
P25679 NAJKA LTCLNCPEMFCGKFQICRNGEKICFKKLHQRRPLS-RYIRGCADTCPVGYPKEMIECCSTDKCNR 5
Q8AY51 BUNCA LTCLICPEKDCQKVHTCRNEEKICVKRFYDKNQLGWRAQRGCAVSCPKAKPNETVQCCSTDKCNK 5
O42255 NAJSP LTCLNCPEMFCGKFQTCRNGEKICFKKLQQRRPFSLRYIRGCAATCPGTKPRDMVECCSTDRCNR 5
O42256 NAJSP LTCLNCPEMFCGKFQTCRDGEKICFKKLQQRRPFSLRYIRGCAATCPGTKPRDMVECCSTDRCNR 5
P29181 NAJNA LTCLNCPEVYCRRFQICRDGEKICFKKFDQRNLLGKRYRRGCAATCPEAKPREIVQCCSTDKCNR 5
Q802B3 NAJSP LTCLNCPEMFCGKFQTCRNGEKICFKMLQQRRPFSLRYIRGCAATCPGTKPRDMVECCSTDRCNR 5
Q802B2 NAJSP LTCLNCPEVFCKKFQTCRNGEKICFKKFDERKLFGKRYRRGCAATCPEAKPREIVQCCSTDRCNR 5
P29179 NAJNA LTCLICPEKYCNKVHTCLNGENICFKRFN--RILGKRYDLGCAATCPTVKT-GIVQCCSTDKCNH 2
P29182 NAJNA LTCLNCPEVYCRRFQKCRNGEKICFKKFDQRNLLGKRYEIGCAATCPEAKPREIVQCCSTDKCNR 5
Q2VBN2 OPHHA LTCLNCPEQYCKRIHTCRNGENVCFKRFYEGKLLCKQFRRGCAATCPEAKSREIVQCCSTDECNH 3
P29180 NAJNA LTCLICPEKYCNKVHTCLNGEKICFKRYSERKLLGKRYIRGCADTCPVRKPREIVQCCSTDKCNH 3
Q8AY50 BUNCA LTCLICPEKDCQKVHTCRNEEKICVKRSYDKNQLGWRAQRGCAVSCPKAKPNETVQCCSTDKCNK 5
Q8AY49 BUNCA LTCLICPEKDCQKVHTCRNEEKICVKRFYDKNQLGWRAQRGCAVSCPKAKPNETVQCCSTDDCNR 4
Q9YGI1 NAJAT LTCLICPEKYCNKVHTCLNGEKICFKKYDQRKLLGKGYIRGCADTCPKLQNRDVIFCCSTDKCNL 3
Q9W7I3 NAJSP LTCLNCPEMFCGKFQTCRNGEKICFKMLQQRRPFSLRYIRGCAATCPGTKPRDMVECCNTDRCNR 5
Q53B61 OPHHA LTCLICPEEYCKRIHTCRDGENVCFKGFYEGKQLGKQFRRGCAATCPEGKPNEIVQCCSTDECNH 2
Q6IZ95 BUNCA LTCLICPEKYCQKVHTCRDGENLCVKRFYEGKRFGKKYPRGCAATCPEAKPHEIVECCSTDKCNK 4
P60814 NAJAT LTCLNCPEMFCGKFQICRNGEKICFKKLHQRRPFSLRYIRGCAATCPGTKPRDMVECCSTDRCNR 5
Q9YGI2 NAJAT LTCLICPEKYCNKVHTCLNGEKICFKKYDQRKLLGKRYIRGCADTCPVRKPREIVECCSTDKCNH 3
```

Non-conventional toxins. Orphan group IV (Candoxin group, strong toxins)

```
P82935 NAJKA LTCLNCPEMFC--GKFQICRNGEKICFKKLHQRRLSWRYIRGCADTCPVGKP-YEMIECCSTDKCNR 5
P81783 BUNCA MKCKICNFDTCRAGELKVCASGEKYCFKESWREARGTRIERGCAATCPKGSVYGLYVLCCTTDDCN- 3
P15818 BUNMU MKCKICHFDTCRAGELKVCASGEKYCFKESWREARGTRIERGCAATCPKGSVYGLYVLCCTTDDCN- 3
P58370 MICCO LECKICNFKTCPTDELRHCASGETICYKTFWNTHRGLRIDRGCAATCPTVK-PGVNIICCKTDNCN- 2
Q70WS8 BUNMU MKCKICHFDTCRAGELKVCASGEKYCFKESWREAREVKIIRGCSSSCPEKK----NVFCCSTNDCNW 3
```

Non-conventional toxins. Orphan group V (γ-BGTx group, inhibit platelet aggregation)

```
P82935 NAJKA LTCLNCPEMFCGKFQICRNGEKICFKKLHQRRP---LSWRYIRGCADTCPVGKPYEMIECCSTDKCNR 5
Q9YGJ0 BUNMU MQCKTCSFYTCPNSETCPDGKNICVKRSWTAVRGDGPKREIRRECAATCPPSKLGLTVFCCTTDNCNH 1
O12963 BUNMU MQCKTCSFYTCPNSETCPDGKNICVKRSWTAVAGDGLKREIRRECAATCPPSKLGLTVFCCTTDNCNH 1
Q9YGH9 BUNMU MQCKTCSFYTCPNSETCPDGKNICVKRSWTAVRGDGPKREIRRECAATCPPSKLGLTVFCCTTDNCYH 1
```

Non-conventional toxins. Orphan group XVII

```
P82935 NAJKA LTCLNCPEMFCGKFQICRNGEKICFKKLHQRRLSWRY----------IRGCADTCPVGKP---YEMIECCSTDKCNR 5
Q8JFX7 BUNMU EMCNMCVRPYPFMSSCCPEGQDRCYKSYWVNENGKQKKYHGKYPVILERGCVTACTGPGSGSIYNLYTCCPTNRCGSSSTSG 2
Q9PW19 BUNMU EMCNMCVRPYPFMSSCCPEGQDRCYKSYWVNENGKQEAYHGKYPVILERGCVTACTGPGSGSIYNLYTCCPTNRCGSSSTSG 2
Q9YGH0 BUNMU EMCNMCVRPYPFMSSCCPEGQDRCYKSYWVNENRKQEAYHGKYPVILERGCVTACTGPGSGSIYNLYTCCPTNRCGSSSTSG 2
```

Non-conventional toxins. Orphan group XIX (Bucandin group)

```
P82935 NAJKA LTCLNCPEMFCGKFQICRNGEKICFKKLHQRR.RYIRGCADTCPVGKP-YEMIECCSTDKCNR 5
C0HJT4 DENAN LECHRRGSFISDGKITCSAKKTFCCKMYEKIFGIYWYGCAKTYTEKNTWNVYSKCCTTNLCNT 3
C0HKZ8 WALAE MECYKCGVSGCHLKITCSAEEKFCYKWRDKISNERWLGCAKTCTEENTWRVYNSCCTTNLCNP 2
P81782 BUNCA MECYRCGVSGCHLKITCSAEETFCYKWLNKISNERWLGCAKTCTEIDTWNVYNKCCTTNLCNT 1
P25682 DENJA LECYRCGVSGCHLRTTCSAKEKFCAKQHNRISTLWWHGCVETCTEDETWKFYRKCCTTNLCNI 3
```

Non-conventional toxins. Boigotoxin group (Strong activity against avian nAChRs)

```
P82935 NAJKA          LTCLNC---PEMFCGKFQICRNGEKICFKKLHQRRLSWRY----IRGCADTCPVGKPYEMIECCSTDKCNR 5
A0S865 BOIIR QAKGPPYTLCFECNRETCSNCFKDNRCPPYHRTCYTLYRPDGNGEM--KWAVKGCAKTCPTAQPGESVQCCNTPKCNDY 4
A0S864 BOIIR QAVGPPYTLCFECNRMTSSDCSTALRC--YRGSCYTLYRPDENCEL--KWAVKGCAETCPTAGPNERVKCCRSPRCNDD 3
C0HJD3 OXYFU QAIGPPFGLCFQCNQKTSSDCFNAKRCPPFHRTCYTLYKPDGG--E--EWAVKGCAKGCPTAGPDERVKCCHTPRCNN- 4
Q06ZW0 BOIDE QAVGLPHGFCIQCNRKTWSNCSIGHRCLPYHMTCYTLYKPDENGEM--KWAVKGCARMCPTAKSGERVKCCTGASCNSD 1
P83490 COERA QAIGPPYGLCFQCNQKTSSDCTEARRCSPFHEKCYTLYQPDENWMKSSGLSHFGCGKQCPTAGPEGRVTCCLTPRCN-- 4
A7X3S2 TRIBI QAIGPPFTRCSKCNRNWSPPFPLGLYTVSHVKSCGGHLSSIGQCGE--DWVVKGCAKTCPTAGPGERVKCCYSPRCNKN 3
A7X3V0 TELDH QAIGPPHGLCVQCDRKTCKNCFKSERCQPYNRICYTLYKPDENGEM--KWAVKGCAKTCPSAKPGERVKCCSSPRCNEV 3
A7X3S0 TRIBI QAIGPPFTRCSQCNRNRSPQCFIEDRCTPGDFTCYTVYKPNGNGGE--DWVVKGCAKTCPTAGPGERVKCCYSPRCNKN 3
```

Short-chain toxins (Type I α-ntx)

```
P82935 NAJKA LTCLNCPEMFCGKFQIC--RNGEKICFKKLHQRR.SWRYIRGC.CPVGKPYEMIECCSTDKCNR 5
ScNTX synth  MICYNQQSSQPPTTKTC--S--ETSCYKKTWRDHRGTIIERGCGCPKVKPGIKLHCCRTDKCNN 3
P60775|LATSE RICFNHQSSQPQTTKTC--SPGESSCYNKQWSDFRGTIIERGCGCPTVKPGIKLSCCESEVCNN 1
```

```
P60770|NAJAT LECHNQQSSQTPTTTGC--SGGETNCYKKRWRDHRGYRTERGCGCPSVKNGIEINCCTTDRCNN   1
P80958|NAJAT LECHNQQSSQTPTTKTC--S-GETNCYKKWWSDHRGTIIERGCGCPKVKPGVNLNCCTTDRCNN   2
P59276|NAJKA LECHNQQSSQAPTTKTC--S-GETNCYKKWWSDHRGTIIERGCGCPKVKPGVNLNCCRTDRCNN   3
Q45Z11|OXYSC MTCYNQQSSEAKTTTTC--SGGVSSCYKKTWSDGRGTIIERGCGCPSVKKGIERICCRTDKCNN   3
C1IC47|WALAE FVCHNQQSSQPPTTTNC--SGGENKCYKKQWSDHRGSITERGCGCPTVKKGIKLHCCTTEKCNN   2
K9MCH1|MICLL RICYNQQSSQPPTTKTC--S--EGQCYKKTWRDHRGTIIERGCACPNVKPGIQISCCTSDKCNG   1
Q7T2I5|LATSE RICFNHQSSQPQTTKTC--SPGESSCYHKQWSDFRGTIIERGCGCPTVKPGINLSCCESEVCNN   1
Q90VW1|LATSE RICFNHQSSQPQTTKTC--SPGESSCYHKQWSDFRGTIIERGCGCPTVKPGIKLSCCESEVCNN   1
P59275|NAJKA LECHNQQSSQTPTTKTC--S-GETNCYKKWWSDHRGTIIERGCGCPKVKPGVNLNCCRRDRCNN   4
P01426|NAJPA LECHNQQSSQPPTTKTC--P-GETNCYKKVWRDHRGTIIERGCGCPTVKPGIKLNCCTTDKCNN   2
P25497|PSEAU MTCCNQQSSQPKTTTIC--AGGESSCYKKTWSDHRGSRTERGCGCPHVKPGIKLTCCKTDECNN   2
P68417|NAJHA LECHNQQSSQPPTTKTC--P-GETNCYKKRWRDHRGSITERGCGCPSVKKGIEINCCTTDKCNN   2
P0CB06|OXYSC MTCYNQQSSEAKTTTTC--SGGVSSCYKKTWSDIRGTIIERGCGCPSVKKGIERICCRTDKCNN   3
P60771|NAJKA LECHNQQSSQTPTTTGC--SGGETNCYKKRWRDHRGYRTERGCGCPSVKNGIEINCCTTDRCNN   1
C1IC48|WALAE LLCHNQQSSTSPTTTCC--SGGESKCYKKRWPTHRGTITERGCGCPTVKKGIELHCCTTDQCNL   1
P86097|MICSU LICYSQMYN--EIIKTC--ENGETTCYSKTWRDHRGTRLEKGCGCPPVKYDMIVKCCKTDRCGN   3
P86095|MICSU MICYNQQSTEPPTTKTC--S--EGQCYKKTWSDHRGTIIERGCACPNVKPGVKISCCSSDKCR-   3
C0HLK3|MICTC MICYNQQSSEPPTTKTC--S--EGQCYKKTWSDHRGTIIERGCACPNVKPGVKISCCSSDK---   1
Q5UFR8|HYDPR MTCCNQQSSQPKTTTNC--A--GNSCYKKTWSDHRGTIIERGCGCPQVKSGIKLECCHTNECNN   1
P68416|HYDHA MTCCNQQSSQPKTTTNC--A--ESSCYKKTWSDHRGTRIERGCGCPQVKPGIKLECCHTNECNN   1
Q9PTT0|NAJNA LECHNQQSSQTPTTTGC--SGGETNCYKKRWRDHRGYRTERGCGCPSVKNGIEINCCTTDRCNN   1
F5CPD8|MICAT MICYNQQSSQPPTTTTC--S--EGQCYKQRWRDHRGWRTERGCGCPKAIPEVKLNCCKTDRCNG   2
P62376|HYDCY MTCCNQQSSQPKTTTNC--A--ESSCYKKTWSDHRGTRIERGCGCPQVKSGIKLECCHTNECNN   1
P01416|DENPO RICYNHQSTTRATTKSC--E--ENSCYKKYWRDHRGTIIERGCGCPKVKPGVGIHCCQSDKCNY   2
P19959|AIPLA LTCCNQQSSQPKTTTDC--A--DNSCYKMTWRDHRGTRIERGCGCPQVKPGIKLECCKTNECNN   2
P01423|NAJNI MICHNQQSSQRPTIKTC--P-GETNCYKKRWRDHRGTIIERGCGCPSVKKGVGIYCCKTDKCNR   4
P82849|NAJKA LECHNQQSSQTPTTTGC--SGGENNCYKKEWRDNRGYRTERGCGCPSVKKGIGINCCTTDRCNN   1
F5CPD5|MICAT MICYNQQSSQPPTTTTC--S--EGQCYKKTWSDHRGTIIERGCACPNVKPGVKISCCSSDKCNG   1
P0CG02|HYDTO MTCCNQQSSQPKTTTNC--A--GNSCYKKTWSDHRGTIIERGCGCPQVKSGIKLECCHTNECNN   1
P86420|MICFR MICYNHQSSEPPTTKTC--S--EGQCYKKSWSDHRGTIIERGCACPNVKPGVKIICCRS--C--   2
P68419|NAJNI LECHNQQSSQPPTTKTC--P-GETNCYKKRWRDHRGSITERGCGCPSVKKGIEINCCTTDKCNN   2
P68418|NAJHH LECHNQQSSQPPTTKTC--P-GETNCYKKRWRDHRGSITERGCGCPSVKKGIEINCCTTDKCNN   2
P01412|OPHHA LICFNQETYRPETTTTC--PDGEDTCYSTFWNDHHGVKIERGCGCPRVNPPISIICCKTDKCNN   2
P10459|LATLA RRCFNHPSSQPQTNKSC--PPGENSCYNKQWRDHRGTITERGCGCPQVKSGIKLTCCQSDDCNN   1
P01431|NAJMO LECHNQQSSEPPTTTRC--SGGETNCYKKRWRDHRGYRTERGCGCPTVKKGIELNCCTTDRCNN   1
P80548|MICNI MICHNQQSSQPPTIKTC--S--EGQCYKKTWRDHRGTISERGCGCPTVKPGIHISCCASDKCNA   2
P86422|MICFR LTCFNDFSPTAHTVEDC--QRGITTCYMKTWRVHRETVIERGCGCPKVKPGIRLKCCTGNTCNY   1
P01427|NAJOX LECHNQQSSQPPTTKTC--S-GETNCYKKWWSDHRGTIIERGCGCPKVKPGVNLNCCRTDRCNN   3
Q9PSN6|NAJSP LECHDQQSSQTPTTTGC--SGGETNCYKKRWRDHRGYRTERGCGCPSVKNGIEINCCTTDRCNN   1
P01421|NAJHA MICYKQRSLQFPITTVC--P-GEKNCYKKQWSGHRGTIIERGCGCPSVKKGIEINCCTTDKCNR   3
P01425|HEMHA LECHNQQSSQPPTTKSC--P-GDTNCYNKRWRDHRGTIIERGCGCPTVKPGINLKCCTTDRCNN   2
P01434|ACAAN MQCCNQQSSQPKTTTTC--PGGVSSCYKKTWRDHRGTIIERGCGCPRVKPGIRLICCKTDECNN   2
P01422|NAJHA MICHNQQSSQPPTIKTC--P-GETNCYKKRWRDHRGTIIERGCGCPSVKKGVGIYCCKTNKCNR   4
P01420|NAJHA MICYKQQSLQFPITTVC--P-GEKNCYKKQWSGHRGTIIERGCGCPSVKKGIEINCCTTDKCNR   3
P01432|NAJMO LNCHNQMSAQPPTTTRC--SRWETNCYKKRWRDHRGYKTERGCGCPTVKKGIQLHCCTSDNCNN   2
F5CPE4|MICAT LICYNYETPLDKTTECC--GNGVTTCFAKSWRDHRGLRTDRGCGCPNVKPGVTINCCKTDRCNG   2
P10457|LATCO RRCYNQQSSQPKTTKSC--PPGENSCYNKQWRDHRGSITERGCGCPTVKPGIKLRCCESEDCNN   2
P01418|DENVI RICYNHQSTTPATTKSC--G--ENSCYKKTWSDHRGTIIERGCGCPKVKRGVHLHCCQSDKCNN   2
P01433|HEMHA LECHNQQSSQTPTTQTC--P-GETNCYKKQWSDHRGSRTERGCGCPTVKPGIKLKCCTTDRCNK   2
P34076|NAJCH MECHNQQSSQPPTTTHC--SGGETNCYEKRWHDHRGTIIERGCGCPTVKPGVKLNCCTTDKCNN   1
P10460|LATLA RRCFNQQSSQPQTNKSC--PPGENSCYRKQWRDHRGTIIERGCGCPTVKPGVKLRCCQSEDCNN   1
P01437|HYDLA MTCCNQQSSQPKTTTNC--A--ESSCYKKTWRDFRGTRIERGCGCPQVKPGIKLECCHTNECNN   1
P68415|HYDSC MTCCNQQSSQPKTTTNC--A--ESSCYKKTWSDHRGTRIERGCGCPQVKPGIKLECCHTNECNN   1
P68412|HYDST MTCCNQQSSQPKTTTNC--A--GNSCYKKTWSDHRGTIIERGCGCPQVKSGIKLECCHTNECNN   1
P60773|NAJPH LECHNQQSSQAPTTKTC--S-GETNCYKKWWSDHRGTIIERGCGCPKVKPGVKLNCCRTDRCNN   3
P25675|NAJHH MICHNQQSSQPPTIKTC--P-GETNCYKKQWRDHRGTIIERGCGCPSVKKGVGIYCCKTDKCNR   4
Q8UW26|HYDHA MTCCNQQSSQPKTTTNC--A--ESSCYKKTWSDHRGTRIERGCGCPQVKHGIKLECCHTNECNN   1
F5CPD9|MICAT LICYNDHGFIGKTTETC--ENGMTTCYEKRWTEARGTRIDRGCGCPNVKPGVNLNCCKTDRCNG   3
P10456|LATCO RRCFNQQSSQPKTTKSC--PPGENSCYNKQWRDHRGSITERGCGCPKVKPGIKLRCCESEDCNN   3
P32879|AIPLA LTCCNQQSSQPKTTTDC--A--DNSCYKKTWQDHRGTRIERGCGCPQVKPGIKLECCKTNECNN   2
P62388|HYDPL MTCCNQQSSQPKTTTNC--A--ESSCYKKTWSDHRGTRIERGCGCPQVKSGIKLECCHTNECNN   1
P34075|NAJAN KICYNQPSSQHPTTKAC--P-GEKNCYRKQWSDHRGTIIERGCGCPTVKPGVKLHCCTTEKCNN   3
P01417|DENJA RICYNHQSTTPATTKSC--G--ENSCYKKTWSDHRGTIIERGCGCPKVKQGIHLHCCQSDKCNN   2
P01419|DENJA RICYNHQSNTPATTKSC--V--ENSCYKSIWADHRGTIIKRGCGCPRVKS--KIKCCKSDNCNL   2
P19958|AIPLA LTCCNQQSSQPKTTTDC--A--DNSCYKKTWKDHRGTRIERGCGCPQVKPGIKLECCKTNECNN   2
P60772|NAJSP LECHNQQSSQAPTTKTC--S-GETNCYKKWWSDHRGTIIERGCGCPKVKPGVKLNCCTTDRCNN   2
P0CAR1|MICPY MICYNQQSSQPPTTKTC--S--EGQCYKKTWSDHRGTISERGCACPNVKPGVKISCCSSDKCNG   2
F5CPD6|MICAT LICYNDHGYIGKTTETC--ENGMTTCYEERWREARGTRIERGCGCYKVKPGVQMNCCKTDRCNG   3
Q8UW27|HYDHA MTCCNQQSSQPKTTTNC--A--ESSCYKKTWSDHRGTRIERGCGCPQVKRGIKLECCHTNECNN   1
P10458|LATCR RRCFNQQSSQPQTNKSC--PPGENSCYRKQWRDHRGTIIERGCGCPTVKPGIKLRCCQSEDCNN   1
P0CAR4|MICPY LICYNDHGYIGETSESC--KNGETTCYEEXWTEARXXIIERACGCXKVKPGVQMKCCKTQ----   2
F5CPD7|MICAT LICYNYWTPLDKTTECC--GNGVTTCFAKSWNDHRGRRTDRGCGCPNVKPGIHLNCCKTDRCNG   3
P60774|NAJSA LECHNQQSSQAPTTKTC--S-GETNCYKKWWSDHRGTIIERGCGCPKVKPGVKLNCCTTDRCNN   2
P14613|NAJKA LECHNQQSIQTPTTTGC--SGGETNCYKKRWRDHRGYRTERGCGCPSVKNGIEINCCTTDRCNN   1
P01424|NAJME MECHNQQSSQPPTTKTC--P-GETNCYKKQWSDHRGTIIERGCGCPSVKKGVKINCCTTDRCNN   2
P68413|HYDOR MTCCNQQSSQPKTTTNC--A--GNSCYKKTWSDHRGTIIERGCGCPQVKSGIKLECCHTNECNN   2
P10808|BUNFA RICLNQQQSTPEDQPTNGQCYIKTDCQNKTWNTHRGSRTDRGCGCPKVKPGINLRCCKTDKCNE   3
P25494|HYDCY MTCCNQQSSQPKTTTNC--A--ESSCYKKTWSDHRGTRIERGCGCPQVKKGIKLECCHTNECNN   1
P25495|LATCR RRCFNHPSSQPQTNKSC--PPGENSCYNKQWRDHRGTIIERGCGCPTVKPGIKLRCCQSDDCNN   1
```

```
P25496|LATCR RRCFNHPSSQPQTNKSC--PPGENSCYNKQWRDHRGTIIERGCGCPQVKSGIKLRCCQSDDCNN         1
P10455|LATCO RRCYNQQSSQPKTTKSC--PPGENSCYNKQWRDHRGSITERGCGCPKVKPGIKLRCCESEDCNN         2
F8J2G5|DRYCN MTCYNQQLSQPQTTTTC--A--ESFCYKKTWS---GTIIERGCGCPPMKPPIRLKCCRTEKCNN         2
F8J2G3|DRYCN MTCYNQQSSQPQTTTTC--A--ESSCYKKTWRDHRGTIIERGCGCPTVKPGIQRVCCATDKCNN         1
Q69CJ8|OPHHA RICLKQEPFQPETTTTC--PEGEDACYNLFWSDHSEIKIEMGCGCPKTEPYTNLYCCKIDSCNK         2
P62389|HYDSC MTCCNQQSSQPKTTTNC--A--ESSCYKKTWSDHRGTRIERGCGCPQVKSGIKLECCHTNECNN         1
P0C555|BUNFA RICLNQQSSEPQTTETC--PNGEDTCYNKTWNTHRGSRTDRGCGCPKVKPGINLRCCKTDKCNQ         2
Q9YGJ5|NAJSP LECHNQQSSQAPTTTGC--SGGETNCYKKSWRDHRGYRIERGCGCPSVKKGIEINCCTTDRCNN         1
P19960|AIPLA LTCCNQQSSQPKTTTDC--A--DDSCYKKTWKDHRGTRIERGCGCPQVKPGIKLECCKTNECNN         2
Q9YGW8|LATLA RRCFNQQSSEPQTNKSC--PPGENSCYNKQWRDHRGTIIERGCGCPQVKSGIKLTCCQSDDCNN         1
Q9YGX1|LATSE RRCFNQQSSEPQTNKSC--PPGENSCYRKQWRDHRGTIIERGCGCPTVKPGIKLRCCESEDCNN         1
A8HDJ7|CRYNI MTCCNQQSSQPKTITTC--A--ESSCYKKTWKDHHGTRIERGCGCPPRKPLIDLICCETDECNN         1
Q9YGC2|LATLA RRCFNHPSSQPQTNKSC--PPGENSCYNKQWRDHRGTIIERGCGCPTVKPGIKLTCCQSEDCNN         1
Q7T2I1|LATLA RRCFNQQSSEPQTNKSC--PPGENSCYRKQWRDHRGTIIERGCGCPTVKPGVKLRCCESEDCNN         1
A8HDJ5|TROCA MTCCNQQSSQPKTTTPC--A--ESSCYKKTWKDNRGTIIERGCGCPNVKPGIDLMCCKTDECNN         2
A8HDJ8|CRYNI MTCCNQQSSQPKTITTC--A--ESSCYKKTWKDHHGTRIERGCGCPKVKPGVGLECCKTDECNN         2
```

Short-chain toxins (Type III α-ntx)

```
P82935 NAJKA LTCLN..GKFQICRNGEKICFKKLHQRRPLSWRYIRGCADTCPVG..IECCSTDKCNR         5
Q9W7K2 PSETE LTCYKGYHDTVVCKPHETICYEYFIPATHGNAILARGCGTSCPGGIRPVCCRTDLCNK         3
Q9W7K0 PSETE LTCYKGYHDTVVCKPHETICYRYLVPATHGNAIPARGCGTSCPGGNHPVCCSTDLCNK         2
Q9W7J7 PSETE LTCYKSLSGTVVCKPHETICYRRLIPATHGNAIIDRGCSTSCPGGNRPVCCSTDLCNK         2
Q9W7J6 PSETE LTCYKRYFDTVVCKPQETICYRYIIPATHGNAITYRGCSTSCPSGIRLVCCSTDLCNK         2
Q9W7K1 PSETE LTCYKGYHDTVVCKPHETICYRYLIPATHGNAIPARGCGTSCPGGNHPVCCSTDLCNK         2
Q9W7J9 PSETE LTCYKGYHDTVVCKPHETICYEYFIPATHGNAILARGCGTSCPGGIRPVCCRTDLSNK         3
A8HDK1 PSETE LTCYKGYHDTVVCKPHETICYEYFIPATHGNVITTRGCSTSCPGGIRPVCCSTDLCNN         1
```

Long-chain toxins (Type II α-ntx)

```
P82935 NAJKA LTCLNCPEMFCGKFQICRNGEKICFKKLHQRRPLS---WRYIRGCADTCPVG-KPYEMIECCSTDKCNR              5
P01391|NAJKA IRCFIT---PDITSKDCPNGH-VCYTKTWCDAFCSIRGKRVDLGCAATCPTV-KTGVDIQCCSTDNCNPFPTRKRP-------- 1
P60615|BUNMU IVCHTT-ATSPISAVTCPPGENLCYRKMWCDAFCSSRGKVVELGCAATCPSK-KPYEEVTCCSTDKCNPHPKQRPG-------- 1
P01379|LATSE RECYLN---P-HDTQTCPSGQEICYVKSWCNAWCSSRGKVLEFGCAATCPSV-NTGTEIKCCSADKCNTYP------------- 1
C0HJD7|DENPO RTCNKT---FSDQSKICPPGENICYTKTWCDAFCSQRGKRVELGCAATCPKV-KAGVEIKCCSTDNCNKFQFGKPR-------- 2
A1IVR8|BUNCA LLCYKT--PSPINAETCPPGENLCYTKMWCDAWCSSRGKVIELGCAATCPSK-KPYEEVTCCSTDKCNPHPKQRPG-------- 1
Q7T3J2|BUNCA IVCHTT-ATSPISAVTCPPGENLCYRKMWCDAFCSSRGKVVELGCAATCPSK-KPYEEVTCCSTDKCNPHPKQRPG-------- 1
P82662|OPHHA LICFIS---S-HDSVTCAPGENVCFLKSWCDAWCGSRGKKLSFGCAATCPKV-NPGIDIECCSTDNCNPHPKLRP--------- 0
P60616|BUNMU IVCHTT-ATSPISAVTCPPGENLCYRKMWCDVFCSSRGKVVELGCAATCPSK-KPYEEVTCCSTDKCNPHPKQRPG-------- 1
P01382|NAJOX ITCYKT--PIPITSETCAPGQNLCYTKTWCDAWCGSRGKVIELGCAATCPTV-ESYQDIKCCSTDDCNPHPKQKRP-------- 0
A1IVR9|BUNCA LLCYKT--PIPINAETCPPGENLCYTKMWCDIWCSSRGKVVELGCAATCPSK-KPYEEVTCCSTDKCNPHPKQRPD-------- 1
P0DKW9|ACAAN VICYVG---YN-NPQTCPPGGNVCFTKTWCDARCHQLGKRVEMGCATTCPKV-NRGVDIKCCSTDKCNPFPKTTPPWKRPRGKP 1
P01384|NOTSC LICYMG----PKTPRTCPRGQNLCYTKTWCDAFCSSRGKVVELGCAATCPIA-KSYEDVTCCSTDNCNPFPVRPRPHP------ 1
P01381|HYDST LSCYLG---Y-KHSQTCPPGENVCFVKTWCDAFCSTRGERIVMGCAATCPTA-KSGVHIACCSTDNCNIYTKWGSGR------- 1
P01380|HYDST LSCYLG---Y-KHSQTCPPGENVCFVKTWCDGFCNTRGERIIMGCAATCPTA-KSGVHIACCSTDNCNIYAKWGS--------- 1
P01388|NAJME IRCFIT---PDVTSQICADGH-VCYTKTWCDNFCASRGKRVDLGCAATCPTV-KPGVNIKCCSTDNCNPFPTRNRP-------- 0
P34074|NAJAN IRCFIT---PRVSSQACPDGH-VCYTKTWCDNFCGINGKRVDLGCAATCPTV-KPGVDIKCCSTDNCNPFPTRKRP-------- 0
Q53B58|OPHHA TKCYVT---PDVKSETCPAGQDICYTETWCDAWCTSRGKRVNLGCAATCPIV-KPGVEIKCCSTDNCNPFPTRKRP-------- 1
P25671|NAJNA IRCFIT---PDITSKDCPNGH-VCYTKTWCDAFCSIRGKRVDLGCAATCPTV-KTGVDIQCCSTDDCDPFPTRKRP-------- 1
Q2VBP8|OPHHA TKCYKT-G-ERIISETCPPGQDLCYMKTWCDVFCGSRGRVIELGCTATCPTV-KPHEQITCCSTDNCNPHPKMKQR-------- 0
P01390|NAJNI IRCFIT---PDVTSQACPDGH-VCYTKMWCDNFCGMRGKRVDLGCAATCPKV-KPGVNIKCCSRDNCNPFPTRKRS-------- 0
P01394|DENVI RTCYKT---PSVKPETCPHGENICYTETWCDAWCSQRGKREELGCAATCPKV-KAGVGIKCCSTDNCDPFPVKNPR-------- 1
P01386|OPHHA TKCYVT---PDATSQTCPDGQDICYTKTWCDGFCSSRGKRIDLGCAATCPKV-KPGVDIKCCSTDNCNPFPTWKRKH------- 0
Q53B53|OPHHA TKCYIT---PDVKSETCPDGENICYTKTWCDVWCGSRGRRVDLGCAATCPIV-KPGVNINCCSTDNCNPFPKRS---------- 1
O42257|NAJSP IRCFIT---PDVTSTDCPNGH-VCYTKTWCDGFCSSRGRRVELGCAATCPTV-KPGVDIQCCSTDNCNPFPTRP---------- 0
P85140|BUNCA IVCHTT-ATSPISAVTCPPGENLCYRKM-CDAICSSRGKVVELGCAATCPSK-KPYEEVTCCSNDKCNPHPKQRPG-------- 1
P01395|DENVI RTCYKT---PSVKPETCPHGENICYTETWCDAWCSQRGKRVELGCAATCPKV-KAGVGIKCCSTDNCNPFPVWNPRG------- 1
Q53B59|OPHHA LICFIS---P-HDSVTCAPGENVCFLKSWCDAWCGSRGKKLSFGCAATCPKV-NPGIDIECCSTDNCNPHPKLRP--------- 0
B2BRQ5|AUSLA LICYMG----PKTPRTCPPGQNLCYTKTWCDGFCGSRGKVVVLGCAATCPTV-KPGVDITCCATDKCNPFPKTKAPWERP---- 3
P01396|DENPO RTCNKT---FSDQSKICPPGENICYTKTWCDAWCSQRGKRVELGCAATCPKV-KAGVEIKCCSTDDCDKFQFGKPR-------- 2
Q9W7J5|PSETE RTCFIT---PDVKSKPCPPGQEVCYTKTWCDGFCGIRGKRVDLGCAATCPTPKKTGIDIICCSTDDCNTFPLRPRGRLSSIKDHP2
P01389|NAJAC IRCFIT---PDVTSQACPDGQNICYTKTWCDNFCGMRGKRVDLGCAATCPTV-KPGVDIKCCSTDNCNPFPTRERS-------- 0
P01383|NAJME KRCYRT---PDLKSQTCPPGEDLCYTKKWCADWCTSRGKVIELGCVATCPKV-KPYEQITCCSTDNCNPHPKMKP--------- 1
P01393|DENJA RTCYKT---YSDKSKTCPRGEDICYTKTWCDGFCSQRGKRVELGCAATCPKV-KTGVEIKCCSTDYCNPFPVWNPR-------- 3
P25667|DENPO RTCNKT---FSDQSKICPPGENICYTKTWCDAWCSRRGKIVELGCAATCPKV-KAGVGIKCCSTDNCNLFKFGKPR-------- 1
P25674|NAJHH IRCFIT---PDVTSQACPDGH-VCYTKMWCDNFCGMRGKRVDLGCAATCPTV-KPGVDIKCCSTDNCNPFPTRKRS-------- 0
P25672|NAJNA IRCFIT---PDITSKDCPNGH-VCYTKTWCDGFCRIRGERVDLGCAATCPTV-KTGVDIQCCSTDDCDPFPTRKRP-------- 1
P25668|NAJNA IRCFIT---PDITSKDCPNGH-VCYTKTWCDGFCSIRGKRVDLGCAATCPTV-RTGVDIQCCSTDDCDPFPTRKRP-------- 1
P14612|PSEAU LTCYKG---RDRSSETCRSEQELCCTKTWCDQWCQDRGPRLEMGCTATCPRR-MPGLDFTCCTTDNCNPVPT------------ 2
P13495|PSETE RTCFIT---PDVKSKPCPPGQEVCYTETWCDGFCGIRGKRVELGCAATCPTPKKTGIDIQCCSTDDCNTFPLR------P---- 2
P01387|OPHHA TKCYVT---PDVKSETCPAGQDICYTETWCDAWCTSRGKRVDLGCAATCPIV-KPGVEIKCCSTDNCNPFPTWRKRP------- 1
P25670|ASPSC RICYIA---P-YDHKTCAAGENICYLKAWCDAWCSSRGKKLEFGCAATCPTV-KPGVDISCCDTDNCNPHPKL----------- 1
P01385|ACAAN VICYRG---YN-NPQTCPPGENVCFTRTWCDAFCSSRGKVVELGCAATCPIV-KSYNEVKCCSTDKCNPFPVRPR------RPP 1
P80965|OPHHA TKCYKT-G-DRIISEACPPGQDLCYMKTWCDVFCGTRGRVIELGCTATCPTV-KPHEQITCCSTDNCDPHHKMLQ--------- 0
P0DQQ1|NAJME KRCYRT---PDLKSQTCPPGEDLCYTKKWCADWCTSRGKVIELGCVATCPTV-KPYEEITCCSTDNCNPHPKMPKP-------- 1
P0DQQ2|NAJME IRCFIT---PDVTSQICADGH-VCYTKTWCDAWCTSRGKRVDLGCAATCPTV-KTGVDIKCCSTDNCNPFPTRNRP-------- 0
P0C8R8|LATCO RICYLA---P-RDTQICAPGQEICYLKSWDDGTGSIRGNRLEFGCAATCPTV-KRGIHIKCCSTDKCNPHPKLA---------- 2
P0C8R7|LATCO RICYLA---P-RDTQICAPGQEICYLKSWDDGTGFLKGNRLEFGCAATCPTV-KPGIDIKCCSTDKCNPHPKLA---------- 2
Q53B54|OPHHA TKCYIT---PDVKSETCPDGENICYTKSWCDVFCTSRGKRIDLGCAATCPKV-KPGVDIKCCSTDNCNPFTPWKRH-------- 1
P0C8R6|LATCO RICYLA---P-RDTQICAPGQEICYLKSWDDGSGSIKGKRLEFGCAATCPTV-KPGIDIKCCSTDKCNPHPKLA---------- 2
```

```
A8HDK6|PSETE LICYLD----FSVPHTCAPGEKLCYTRTWNDG----RGTRIERGCAATCPIPKKPEIHVTCCSTDRCNPHPKQKPH-------- 2
A1IVR7|BUNCA LLCYKT--PSPINAETCPPGENLCYTKMWCDAWCSSRGKVVELGCAATCPSK-KPYEEVTCCSTDKCNPHPKQRPD-------- 1
P07526|OPHHA TKCYVT---PDVKSETCPAGQDLCYTETWCVAWCTVRGKRVSLTCAAICPIV-PPKVSIKCCSTDACGPFPTWPNVR------- 1
P01397|DENPO RTCNKT---PSDQSKICPPGENICYTKTWCDAWCSQRGKIVELGCAATCPKV-KAGVEIKCCSTDNCNKFKFGKPR-------- 2
F8J2F2|DRYCN FSCYKT---PYVKSEPCAPGENLCYTKSWCDRFCSIRGKVIELGCAATCPPA-EPKKDITCCSTDNCNTHP------------- 1
D2N116|BUNCE LLCHTT-STSPISTVTCPSGENLCYTKMWCDAFCSSRGKVIELGCVATCPQP-KPYEEVTCCSTDKCNPHPKQRPG-------- 1
A6MFK5|DEMVE RTCLKT---PEVKSEPCPPGQEVCYTKAWRDRMCSFRGKVIELGCAATCPRQ-EPGKEITCCSTDDCNTHP------------- 1
P25669|NAJNA IRCFIT---PDITSKDCPNGH-VCYTKTWCDGFCSSRGKRVDLGCAATCPTV-RTGVDIQCCSTDDCDPFPTRKRP-------- 1
P34073|ACAAN VICYRK---YTNNVKTCPDGENVCYTKMWCDGFCTSRGKVVELGCAATCPIR-KPGNEVKCCSTNKCNHPPKRKKRR------P 2
P25673|NAJNA IRCFIT---PDITSKDCPNGH-VCYTKTWCDGFCSRRGERVDLGCAATCPTV-KTGVDIQCCSTDDCDPFPTRKRP-------- 1
F8J2D7|DRYCN RKCYKT---HPYKSEPCAPGENLCYTKTWCDFRCSQLGKAVELGCAATCPTT-KPYEEVTCCSTDDCNRFPNWERPRPRPR..  2
C5ILC5|OPHHA LLCYKT--PSPINAETCPPGENLCYTKMWCDAWCSSRGKVIELGCAATCPSK-KPYEEVDCCSTDNCNPHPKLRP--------- 0
F8J2E5|DRYCN LICYLG----YNNPQTCAPGQNLCYTKKWCDAFCLQRGKVIQLGCAATCPTT-KPYEEVTCCSRDKCNPHPAQRSR-------- 1
Q2VBP4|OPHHA LICFIS---S-HDSVTCAPGENVCFLKSWCDAWCGSRGKKLSFGCAATCPRV-NPGIDIECCSTDNCNPHPKLRP--------- 0
Q8UW28|HYDHA LSCYLG---Y-KRSQTCPPGEKVCFVKSWCDAFCGSRGKRIEMGCAATCPTV-KDGIDITCCATDNCNTYANWGSG-------- 3
F8J2E6|DRYCN FSCYKT---PYVKSEPCAPGENLCYTKSWCDAFCSIRGKVIELGCAATCPPA-EPKKDITCCSTDNCNTHP------------- 1
F8J2B3|DRYCN RKCYKT---HPYKSEPCASGENLCYTKTWCDFRCSQLGKAVELGCAATCPTT-KPYEEVTCCSTDDCNRFPNWERPRPRPR..  2
Q2VBP5|OPHHA TKCYVT---PDVKSETCPDGENICYTKSWCEVFCTSRGKRIDLGRAATCPKV-KPGVDIKCCSTDNCNPFTPWKRH-------- 1
F8J2E2|DRYCN LICYMG----PKTPRTCPPGENLCYTKTWCDAFCSIRGRRVDLGCAATCPTA-KPGVDITCCSTDKCNPHPAHQSR-------- 3
Q53B57|OPHHA TKCYVT---PDVTSQTCPDGQNICYTETWCDAWCGSRGKRVNLGCAATCPKV-NPGVDIICCSTDNCNPFPKRS---------- 0
F8J2E1|DRYCN LICYQA----YNTPQTCAPGENLCYTKTWCDYWCHVKGKRIDLGCAATCPTA-KPGEDVTCCSRDKCNPHPLQRPR-------- 2
A8S6B0|AUSSU LICYVD----SKTSRTCPPGENVCFTETWCDARCSLLGKRVDLGCAATCPTA-KPGVDITCCSTDKCNPFPTQKHR-------- 3
B2BRQ6|AUSLA LRCYMG----PKTPRTCPPGENLCFTKTWCDPRCSLLGKLVKLGCAATCPIP-KSYEDVTCCSTDNCNRFPKWERSRPRP..   3
A6MFK4|DEMVE RTCLKT---PEVKSEPCPPGQEVCYTKAWCDRMCSFRGKVIELGCAATCPRQ-EPGKEITCCSTDDCNTHP------------- 1
P80156|OPHHA TKCYKT-G-DRIISEACPPGQDLCYMKTWCDVFCGTRGRVIELGCTATCPTV-KPHEQITCCSTDNCNPHPKMKQ--------- 0
Q2VBP3|OPHHA TKCYKT-G-ERIISETCPPGQDLCYMKTWCDVFCGSRGRVIELGCTATCPTV-KHHEQITCCSTDNCNPHPKMKQR-------- 0
Q2VBP6|OPHHA TKCYKT-G-ERIISETCPPGQDLCYMKTWCDVFCGSRGRVVELGCTATCPTV-KPHEQITCCSTDNCNPHPKMKQR-------- 0
Q53B56|OPHHA RICHKS---S-FISETCPDGQNLCYLKSWCDIFCGSRGERLEFGCAATCPEV-KPGVNIECCSTDNCNPHPKLRP--------- 0
A8HDK4|TROCA FSCYKT---PHVKSEPCAPGQNLCYTKTWCDAFCFSRGRVIELGCAATCPPA-EPKKDISCCSTDNCNPHPAHQSR-------- 1
A8HDK9|OXYSC RRCFTT---PSVRSERCPPGQEVCYTKTWTDGHGGSRGKRVDLGCAATCPTPKKKDIKIICCSTDNCNTFPKW------P---- 2
A8S6A8|AUSSU FSCYKT---PDVKSEPCAPGENLCYTKTWCDRFCSIRGKVIELGCAATCPPA-EPRKDITCCSTDNCNPHPAH----------- 1
A7X4R0|OXYMI RRCFTT---PSVRSERCPPGQEVCYTKTWTDGHGGSRGKRVDLGCAATCPTPKKKDIKTICCSKDNCNTFPKW------P---- 3
Q7T2I3|LATLA RICFKT---PYVKSETCPPGQELCYTKTWCDRFCSIRGKVIELGCTATCPRA-EPKEDTTCCSKDNCNPHP------------- 2
A8N285|OPHHA TKCYVT---PDATSQTCPDGENICYTKSWCDVFCSSRGKRIDLGCAATCPKV-KPGVDIKCCSTDNCNPFTPWKRH-------- 0
A8HDK8|OXYMI RRCFIT---PDVRSERCPPGQEVCYTKTWCDGFCSSRGKRVDLGCAATCPTPKKKGIDIICCSKDNCNTFPKW------P---- 3
A7X4Q3|OXYMI RRCFIT---PDVRSERCPPGQEVCYTKTWCDGFCGSRGKRVDLGCAATCPTPKKKGIDIICCSKDNCNTFPKW------P---- 3
A3FM53|HYDHA RTCYRT---HPYKPETCPPGQNLCYKKSWCDAFCSSRGKVIELGCTAKCPTV-KHGKDINCCATDNCNTVANWKSR-------- 1
A8HDK7|OXYMI RRCFIT---PDVRSERCPPGQEVCYTKTWCDGFCGSRGKRVDLGCAATCPTPKKKDIKIICCSKDNCNTFPKW------P---- 3
Q8UW29|HYDHA RTCFRT----PYKPETCPPGQNLCYKKSWCDAFCSSRGKVIELGCTAKCPTV-KDGKDITCCATDNCNTVANWKSR-------- 1
```

Supplementary Fig. 11. Amino acid alignment of the sequences of non-conventional, short-chain, and long-chain snake neurotoxins. The sequence data were obtained from the UNIPROT Database. Only reviewed entries were used. The WTX sequence (P82935) is shown above each group. Cysteine residues are highlighted by yellow background and region responsible for the WTX membrane activity (second half of the loop I, head-1, and C-terminal fragment) is in gray. The number of positively charged residues (Arg and Lys) in the possible membrane-active site of the toxins is calculated and shown on the right. The toxins having 3, 4, and 5 positively charged residues are highlighted by blue, green, and red colors, respectively. Classification of neurotoxins proposed by Fry et al [3] is used.

α7-nAChR/epibatidine+WTX

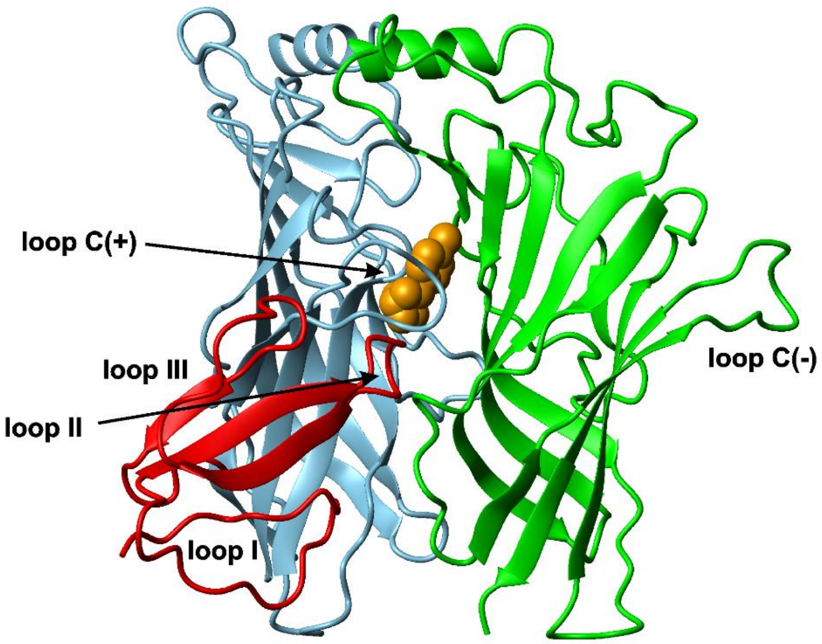

Supplementary Fig. 12. Structure of the α7-nAChR/epibatidine complex (PDB ID: 7KOX [2]**) with superimposed the WTX molecule from the final α7-nAChR/WTX solution after MD.**

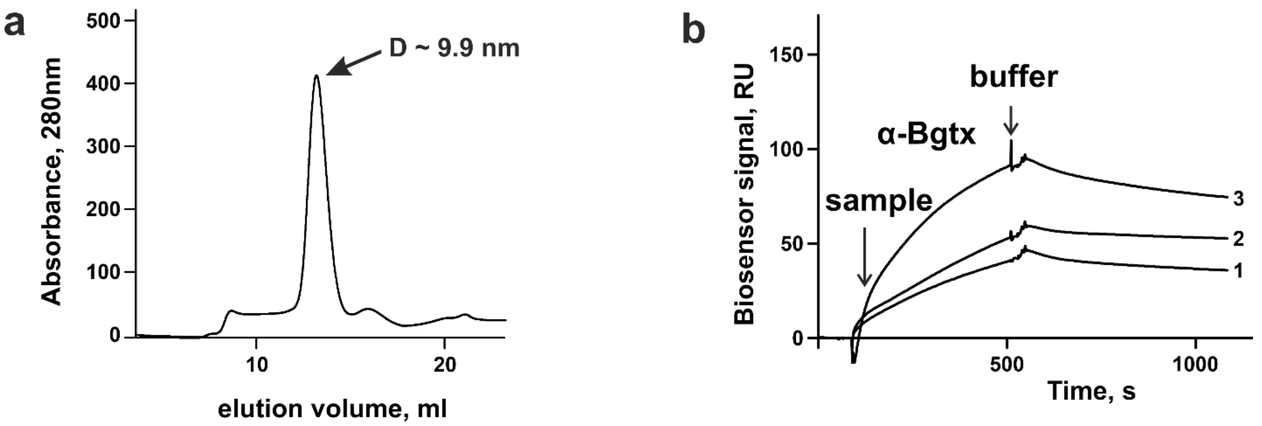

Supplementary Fig. 13. **Characterization of α7-ECD**. **a** SEC analysis of α7-ECD (Superdex-200, 10/300, GE Healthcare). **b** SPR analysis of α7-ECD interaction with α-Bgtx. Representative sensorgrams of α7-ECD injection into the biosensor channels (SPR biosensor Biacore 8k, GE Healthcare) at 25°C with immobilized α-Bgtx. Sensorgrams were corrected to the baseline channel signal without immobilized ligand. Domain concentration in injections 1, 2, 3 was 250, 550, and 1200 nM, respectively. Arrows indicate the start and finish of the α7-ECD injection. Determined K_d value is about 26 nM.

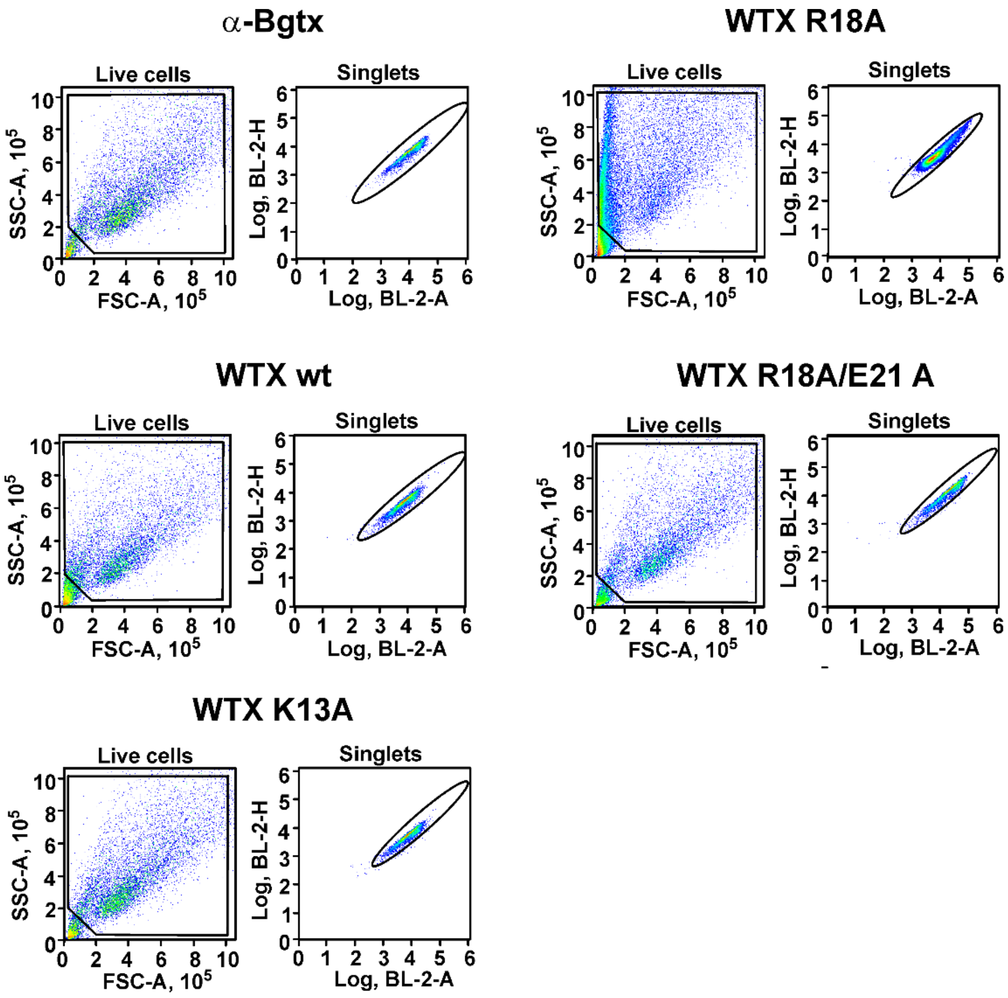

Supplementary Fig. 14. The gating strategy used for binding studies. The side (SSC) versus forward (FSC) scattering plots used for identification of live cells are shown on the left panels, the height (BL-2-H) versus amplitude (BL-2-A) plots used for identification of cell singlets are shown on the right panels.

Supplementary Tables

Supplementary Table 1. Summary of the toxin-receptor interactions in MD trajectory of α7-nAChR/WTX complex.

WTX residue [a)]	α7-nAChR, residue [b)]	
	**primary (+) subunit**	**complementary (-) subunit**
**loop I**		
P7		P192 (M)
M9	Y151 (M)	I191 (M); P192 (M); N193 (H)
F10	Y151 (S, M); I152 (M)	
C11	Y151 (M)	
**loop II**		
L28	F209 (M)	
R31	F209 (π, S)	
R32	E211 (H, I);	W77 (π, S); D186 (H, I); S188 (H)
A33	W171 (M); F209 (M); Y210 (M); C212 (M)	L60(M); W77(M)
L34	W171 (M); Y115 (M); Y210 (M)	L60 (M); W77 (M); L141 (M)
W36	Y115 (M); K167 (π); W171 (M); R208 (π, S); Y210 (S)	L60 (M); I191 (M); P192 (M)
R37		P192 (H)
Y38	K165 (π)	P192 (M)
I39		P192 (M)
**loop III**		
P51	F209 (M)	
Y52	F209 (S, M); Y210(M); P216 (M); Y217 (M)	
M54	F209 (M); Y217 (M)	
E56	K204 (H, I); S206 (H)	
***C*-terminus**		
K62	D160 (H, I); E495 (H, I)	
R65	D153 (I); D160 (H, I); H163 (π, S); K165 (H, I [c)])	

a) The WTX[P33A] mutant was used in the computer modelling study. The recombinant toxin and its mutants contain the additional *N*-terminal Met0 residue appearing due to translation of starting *atg* codon.

b) H, S, I, π, and M in brackets denote the types of interaction: hydrogen bond, stacking, ionic bridge or ion–dipole interaction, π-cation interaction, and hydrophobic contact (according to molecular hydrophobicity potential), respectively. The H, S, I, and π contacts are included in this table, if they exist > 10% of time in the 300-ns MD trajectory (at average per one of five WTX instances) and are encountered in at least two of five WTX instances. The M contact is included in the table, if it exists > 40% of time and is encountered in at least four of five WTX instances. Hydrophobic contacts between non-polar residues only were taken into account.

c) The ionic bridge with the *C*-terminal carboxyl group is formed.

Supplementary Table 2. Summary of the toxin-lipid interactions in MD trajectory of α7-nAChR/ WTX complex.

WTX residue [a)b)]	POPC+POPE				Cholesterol	
	Ethano lamine	Carbo nyl	PO_4^-	Fatty tails	OH	Hydro phobic part
***N*-terminus**						
M0	H	H, I [c)]	H, I [c)]	M		M
L1				M		M
T2			H	M	H	M
**loop I**						
C3				M		M
L4				M		M
N5			H			
C6				M		M
P7				M		
E8	H, I					
G12	H					
K13		H, I	H, I		H	
F14	π		H	M	H	M
Q15		H	H		H	

WTX residue	POPC+POPE				Cholesterol	
	Ethano lamine	Carbo nyl	PO_4^-	Fatty tails	OH	Hydro phobic part
**head-1**						
I16			H	M	H	M
C17				M		
R18	H	H, I	H, I		H	
N19		H	H		H	
G20			H			
E21	H, I					
K22			H, I			
**head-2**						
C42	H					
***C*-terminus**						
T60			H			
D61	H, I					
K62		H, I	H, I			
R65			H, I			

a) The WTX[P33A] mutant was used in the computer modelling study. The recombinant toxin and its mutants contain additional the *N*-terminal Met0 residue appearing due to translation of starting *atg* codon.

b) H, I, π, and M denote the types of interaction: hydrogen bond, ionic bridge or ion–dipole interaction, π-cation interaction, and hydrophobic contact (according to molecular hydrophobicity potential), respectively. The contacts are included in this table, if they exist > 10% of time in the 300-ns MD trajectory.

c) The ionic interactions with the *N*-terminal amino group are observed.

Supplementary Table 3. Parameters of the WTX and its mutants interaction with lipid vesicles POPC/POPG/CHOL (7:1:2) obtained using the Langmuir isotherm. [a)]

Toxin	K_N , μM [a)]	N [a)]
WTX	12±2	28±2
WTX[K13A]	19±1	28 [b)]
WTX[R18A]	40±3	28 [b)]
WTX[R18A,E21A]	140±8	28 [b)]

[a)] $K_N = [P]_{free} \cdot (0.6 \cdot [Lipid] / N - [P]_{bound}) / [P]_{bound}$,
where $[P]_{bound}$ is concentration of the peptide bound to the lipid vesicles ($[P]_0 = [P]_{free} + [P]_{bound}$);
K_N – the equilibrium dissociation constant of the complex between the toxin and the binding site on the vesicle surface;
N – number of lipids forming the binding site on the vesicle surface;
0.6 · [Lipid] – lipid concentration in the outer leaflet of unilamellar vesicles.
Errors represent standard error for parameters estimates (errors of the fit)

[b)] This parameter was fixed in analogy with wild-type WTX.

Supplementary Table 4. Systems setup for MD calculations.

System	Box dimensions$^{\&}$, nm	# of atoms	MD length, ns	# of trajectories
**Equilibrium MD**				
WTX/<br>Water*/Cl$^{-}_{6}$<br><br>**Number of water molecules in 4 starting configurations:*<br>*9556, 8326, 9518, 8573*	*4 starting configurations*<br>6.67×6.67×6.67<br>6.39×6.39×6.39<br>6.66×6.66×6.66<br>6.44×6.44×6.44	 29 729<br>26 039<br>29 615<br>26 780	200	4
α7-AChBP/Water$_{39084}$/Na$^{+}_{5}$	10.97×10.97×10.97	133 567	200	1
α7-nAChR/WTX$_{5}$/<br>POPC$_{449}$/POPE$_{231}$/Cholesterol$_{247}$/<br>Water$_{113343}$/Na$^{+}_{10}$	15.48×15.48×19.69	484 978	300<br>100	1<br>4
**Umbrella sampling**			***relaxation/ production:***	***# of umbrella configurations:***
α7-nAChR/WTX/<br>POPC$_{544}$/POPE$_{269}$/Cholesterol$_{279}$/<br>Water$_{180879}$/Na$^{+}_{34}$	24.15×12.07×23.60	703 238	0.3/1	76
WTX/<br>POPC$_{254}$/POPE$_{128}$/Cholesterol$_{128}$/<br>Water$_{66529}$/Cl$^{-}_{6}$	10.82×10.82×21.69	260 156	0.3/1	60
α7-ECD/WTX/<br>Water$_{106471}$/Na$^{+}_{326}$/Cl$^{-}_{312}$	11.85×23.70×11.85	337 871	0.3/2	67
α7-ECD/α-BgTX/<br>Water$_{106510}$/Na$^{+}_{330}$/Cl$^{-}_{312}$	11.86×23.7×11.86	337 981	0.3/2	66

$^{\&}$The boxes dimensions for last frames of equilibrium MD are indicated.

Supplementary References

[1] S. Huang, S.-X. Li, N. Bren, K. Cheng, R. Gomoto, L. Chen, S.M. Sine, Complex between α-bungarotoxin and an α7 nicotinic receptor ligand-binding domain chimaera, Biochem. J. 454 (2013) 303–310. https://doi.org/10.1042/BJ20130636.

[2] C.M. Noviello, A. Gharpure, N. Mukhtasimova, R. Cabuco, L. Baxter, D. Borek, S.M. Sine, R.E. Hibbs, Structure and gating mechanism of the α7 nicotinic acetylcholine receptor, Cell. 184 (2021) 2121-2134.e13. https://doi.org/10.1016/j.cell.2021.02.049.

[3] B.G. Fry, W. Wüster, R.M. Kini, V. Brusic, A. Khan, D. Venkataraman, A.P. Rooney, Molecular evolution and phylogeny of elapid snake venom three-finger toxins, J. Mol. Evol. 57 (2003) 110–129. https://doi.org/10.1007/s00239-003-2461-2.
